# An innovative approach to the assessment of hydro-political risk: A spatially explicit, data driven indicator of hydro-political issues

**DOI:** 10.1016/j.gloenvcha.2018.07.001

**Published:** 2018-09

**Authors:** F. Farinosi, C. Giupponi, A. Reynaud, G. Ceccherini, C. Carmona-Moreno, A. De Roo, D. Gonzalez-Sanchez, G. Bidoglio

**Affiliations:** a European Commission, DG Joint Research Centre, Ispra, Italy; b Toulouse School of Economics - National Institute for Research in Agriculture (INRA) – University of Toulouse Capitole, Toulouse, France; c Department of Economics, Venice Centre for Climate Studies (VICCS), Ca’ Foscari University of Venice, Venice, Italy

**Keywords:** Hydro-political risk, Water cross-border issues, Transboundary water interactions, Random Forest regression

## Abstract

Competition over limited water resources is one of the main concerns for the coming decades. Although water issues alone have not been the sole trigger for warfare in the past, tensions over freshwater management and use represent one of the main concerns in political relations between riparian states and may exacerbate existing tensions, increase regional instability and social unrest. Previous studies made great efforts to understand how international water management problems were addressed by actors in a more cooperative or confrontational way. In this study, we analyze what are the pre-conditions favoring the insurgence of water management issues in shared water bodies, rather than focusing on the way water issues are then managed among actors. We do so by proposing an innovative analysis of past episodes of conflict and cooperation over transboundary water resources (jointly defined as “hydro-political interactions”). On the one hand, we aim at highlighting the factors that are more relevant in determining water interactions across political boundaries. On the other hand, our objective is to map and monitor the evolution of the likelihood of experiencing hydro-political interactions over space and time, under changing socioeconomic and biophysical scenarios, through a spatially explicit data driven index. Historical cross-border water interactions were used as indicators of the magnitude of corresponding water joint-management issues. These were correlated with information about river basin freshwater availability, climate stress, human pressure on water resources, socioeconomic conditions (including institutional development and power imbalances), and topographic characteristics. This analysis allows for identification of the main factors that determine water interactions, such as water availability, population density, power imbalances, and climatic stressors. The proposed model was used to map at high spatial resolution the probability of experiencing hydro-political interactions worldwide. This baseline outline is then compared to four distinct climate and population density projections aimed to estimate trends for hydro-political interactions under future conditions (2050 and 2100), while considering two greenhouse gases emission scenarios (moderate and extreme climate change). The combination of climate and population growth dynamics is expected to impact negatively on the overall hydro-political risk by increasing the likelihood of water interactions in the transboundary river basins, with an average increase ranging between 74.9% (2050 – population and moderate climate change) to 95% (2100 - population and extreme climate change). Future demographic and climatic conditions are expected to exert particular pressure on already water stressed basins such as the Nile, the Ganges/Brahmaputra, the Indus, the Tigris/Euphrates, and the Colorado. The results of this work allow us to identify current and future areas where water issues are more likely to arise, and where cooperation over water should be actively pursued to avoid possible tensions especially under changing environmental conditions. From a policy perspective, the index presented in this study can be used to provide a sound quantitative basis to the assessment of the Sustainable Development Goal 6, Target 6.5 “Water resources management”, and in particular to indicator 6.5.2 “Transboundary cooperation”.

## Highlights

• Data driven spatially explicit index of hydro-political issues magnitude.
• Estimation of the non-linear interactions between factors determining water issues.
• Increasing climate change and population are likely to boost hydro-political issues.

## 1 Introduction

Future availability of freshwater for human consumption under a changing world represents one of the main concerns of the current political debate. Water crises have been placed among the major risk factors for the coming decades by the Global Risks Perception Surveys conducted by the World Economic Forum between 2015 and 2017 (WEF, 2017, 2016). Increasing demographic pressure, environmental degradation, and climate change impacts on water spatio-temporal distribution represent the largest determinants of current and future water related issues. Although it is intuitive that water stress is likely to increase the competition over water (Malthus, 1798), it is not completely clear how the combinations of factors influencing water demand and availability alone could lead to such different outcomes in different watersheds spread around the planet. Evidence shows that the consequences of comparable levels of physical water stress have been handled unevenly in different geographical areas and historical contexts (Wolf et al., 2003). Socioeconomic and cultural characteristics (Wolf, 2009), jointly with topographic factors (Beck et al., 2014; Gleditsch et al., 2006; Munia et al., 2016), were identified as the drivers more likely influencing hydro-political dynamics. Resource scarcity is likely to increase tensions, especially when associated with socio-cultural stressors (Sirin, 2011), but, on the other hand, the lack of a vital resource as water is also likely to boost cooperation between actors sharing the same freshwater sources (Bernauer et al., 2012b; Wolf, 2009, 2007; Wolf et al., 2003). The literature hardly identified common features between countries involved in water issues: similar levels of tension over water arose between countries independently of their climate zone, population size, territorial extension, level of democracy (Wolf, 2009). Moreover, the same international water issue frequently resulted in episodes of conflict and cooperation at the same time (Gerlak and Zawahri, 2009; Kalbhenn and Bernauer, 2012; Wolf, 2009; Wolf et al., 2003; Yoffe et al., 2004; Zeitoun et al., 2011; Zeitoun and Mirumachi, 2008). Although several cases of tensions, mostly non-violent, were also recorded, the literature shows that water related issues are more likely to be resolved with cooperation between the countries sharing the transboundary basins (De Stefano et al., 2010b; Wolf, 2009,^2007^; Wolf et al., 2003; Yoffe et al., 2004, 2003). Analyzing historical events, Böhmelt et al. (2014) concluded that physical availability and water demand components are only part of the aspects to be considered for the analysis of water related issues. The literature about political science, geopolitics, and diplomacy showed that also socioeconomic factors, jointly with institutional capacity, legal framework, and cultural background influence the diplomatic interactions between countries or actors sharing resources (Bernauer et al., 2012b; Wolf, 2009; Zeitoun et al., 2011) (1).

The goal of this study is to design an empirically based index aimed at analyzing and mapping the interactions between biophysical and socioeconomic factors linked to water issues at global scale. This was done analyzing water availability and demand, as well as socioeconomic, institutional, legal, and cultural context: factors that are likely to influence transboundary water issues. Final goal is to provide the policy maker with an instrument able to capture historical and current determinants of water related issues, but also the possibility to construct scenarios and simulate sets of policy options. The hereby presented index was calculated by applying a machine learning model on data layers at detailed spatial resolution for the assessment of water related issues and their determinants in the interactions between countries in transboundary basins.

### 1.1 Assessing the factors influencing water cross-border issues

#### 1.1.1 From water conflict and cooperation events to water interactions

Political debate at the highest level had often expressed the concern for an increasing number of violent conflicts related to water resources use and appropriation, in particular in the cases of transboundary basins. Such concern brought to the inclusion in Agenda 2030 of a specific indicator on “Proportion of transboundary basin area with an operational arrangement for water cooperation”^2^ (6.5.2), together with “Degree of integrated water resources management implementation” (6.5.1), for the assessment of Target 6.5 “Water resources management”. Nevertheless, the analytic evidence of the correlation between violent conflicts and climatic factors is not completely clear (Buhaug, 2010; Kallis and Zografos, 2014; Zeitoun and Mirumachi, 2008), and thus the need emerges for methods oriented to pursue a scientifically sound and quantitative assessment of available information, as the one proposed herein.

The literature found a strong correlation between temperature (Burke et al., 2009), or drought events (Couttenier and Soubeyran, 2014), and civil war episodes in Africa. Buhaug (2010) firmly contested these findings and found the conflicts to be explained by structural and contextual conditions, such as: exclusion of ethnical groups from the political context, poor economic management, and geopolitical dynamics. Hsiang et al. (2011) proposed a meta-analysis based on 60 studies focusing on 45 historical conflicts on a global scale concluding that temperature and rainfall variability are significantly connected to violent events. Water related issues follow different dynamics respect to civil conflicts: historical water crises were often resolved with more or less satisfactory, formal or informal, agreements between the parties (De Stefano et al., 2010b). Water conflicts in history are, in fact, peripheral events and none of them reached a formal declaration of war (Böhmelt et al., 2014; Kalbhenn and Bernauer, 2012; Katz, 2011; Wolf, 1998, 2007, Yoffe et al., 2004, 2003). The fact that water war episodes were not recorded in the past does not imply that this could not happen in the future (Kallis and Zografos, 2014). Water related disputes were sometimes identified as igniting factors exacerbating international issues of different nature (Wolf, 2009). On the other hand, cooperation over transboundary basins often resulted in a benefit multiplier opportunity, associated with lower costs, increasing benefits and possibility for cooperation beyond water (Sadoff and Grey, 2002). In the analysis of historical hydro-political events, research points out that certain degrees of conflict and cooperation coexists in the same water related event (Gerlak and Zawahri, 2009; Kalbhenn and Bernauer, 2012; Wolf, 2009; Wolf et al., 2003; Yoffe et al., 2004; Zeitoun et al., 2011; Zeitoun and Mirumachi, 2008). For this reason, some authors (in particular Zeitoun and Mirumachi, 2008) claimed it would be more appropriate to analyze the transboundary water interactions, conflict and cooperation dynamics within the same water issue, regardless of their nature (Kallis and Zografos, 2014; Watson, 2015; Zeitoun and Mirumachi, 2008). In the proposed study, this approach was adopted focusing on the historical water interactions, rather than on the specific conflict or cooperation events linked with each of the water related transboundary issues, and use this as an indicator of the hydro-political risk, not intended as conflict risk, but rather risk of experiencing water related issues. As specified in Kalbhenn and Bernauer (2012), each water case underlying the interactions is defined as a water management issue that manifests in multiple interrelated interactions. For instance, the construction of a dam could represent a water case, while the protests of the downstream countries, of the affected stakeholders, the negotiations, and a possible international agreement would represent a series of events (conflict and cooperation) related to the specific case of the construction of our dam. Following Wolf et al. (2003 and 2009), conflictive and cooperative events were defined water interactions. In this paper, we will refer to the water interactions irrespectively of their specific nature and to more generic water issues or cases, defined as the water management aspects determining the interconnected water interactions, as for Wolf et al. (2003) and Yoffe et al. (2003). The likelihood of having water interactions is an indicator of the complexity of the underlying water issue that, if not properly and promptly addressed by the actors involved, could eventually increase the hydro-political risk. Therefore, our index should then be interpreted as a measure of the magnitude of water issues between specific actors in a specific basin. The rationale behind this is the following: if there are interactions about shared water resources in a specific basin, both in the case of tension or cooperation events, there is a water allocation/management/quality issue. Therefore, the fact that a water management issue leads to cooperative or conflictive behaviors is unrelated to the nature of the water issue itself. It attains more to the political, cultural, institutional, and socioeconomic conditions of the actors involved. The presence of a water issue is in itself an indicator of risk: it is a *necessary* condition for having water interactions and a *not sufficient* condition for either conflict or cooperation over water or both. Some water interactions end up being conflictive, some others cooperative, but all imply the existence of a water issue. This study focuses on the analysis of the probability of having water issues, their intensity, and their determinants: the necessary conditions for ensuing water tensions or cooperation. The analysis of the factors that makes the water issue being managed with a more confrontational or cooperative approach by the actors involved falls outside the scope of this research.

#### 1.1.2 Determinants of cross-border water issues

Economic, statistic and game theory approaches have been used to analyze international dynamics over transboundary waters (Dinar, 2004). Some studies analyzed the dynamics of conflict and cooperation, here defined water interactions (Bernauer and Böhmelt, 2014; Böhmelt et al., 2014; Brochmann and Gleditsch, 2012; De Stefano et al., 2012, 2010b; Wolf, 2007; Wolf et al., 2003); other focused on the likelihood of reaching bi- or multi-lateral agreements between countries (Dinar et al., 2011; Espey and Towfique, 2004; Zawahri and Mitchell, 2011); additional analyses used the existence of treaties and River Basin Organizations (RBO’s) as proxy to quantify the institutional resilience toward potential hydro-political tensions (De Stefano et al., 2017, 2012; Petersen-Perlman, 2016). The likelihood of cooperating and finally reaching water agreements is influenced by time-invariant factors, as for geographical and topographic characteristics, and time-varying correlates, as for climatic variables and socioeconomic characteristics. Quantitative analysis was used to find the causal relations leading to conflicting or cooperative interactions and the formation of treaties. Wolf (2003, 2007, and 2009) underlined the central role of the quality, stability and strength of the institutions, highlighting the need for stronger institutional frameworks to cope with future challenges (Giordano and Wolf, 2003). Zawahri and Mitchell (2011) argued that the formation of treaties is a by-product of state interest, transaction costs, and distribution of power. Dinar et al. (2011) analyzed the main reasons why some treaties would be more likely discussed in some basins relative to others. They found that scarcity and cooperation follow an inversed U-shaped curvilinear relation: cooperation is higher when water scarcity is moderate, instead of very high/low (also in Dinar et al., 2010). Extreme scarcity situations were found to be inhibiting factors (Dinar et al., 2011). Institutional stability and effective past agreements oriented toward a fair and efficient water allocation between riparians were found to be cooperation boosting factors (Dinar et al., 2015). These and other studies (Beck et al., 2014; Brochmann and Gleditsch, 2012; Espey and Towfique, 2004) found evidence of the influence of economic factors, trade dependency, virtual water trade, presence of water infrastructures, quality of the institutions, governance, presence of supra-national authorities, cultural background, on the bi- and multi-lateral relations between the countries facing allocation, management, and pollution problems over shared water. A large part of these analyses highlighted the non-linear nature of the relations between water interactions and correlated factors.

In this study, we propose for the first time the use of a machine learning approach to quantitatively assess the linear and non-linear relations between the hydro-political interactions recorded and the time-varying and time-invariant biophysical, topographic, and socioeconomic explanatory variables. We aim at combining information at transboundary river basin level with gridded data into an empirically based data driven index. A similar objective was pursued in the AQUEDUCT Water Risk Atlas developed by the World Resources Institute (WRI) (Gassert et al., 2014, 2013). AQUEDUCT did not specifically refer to hydro-political risk, but rather to a global database of 12 main indicators about water quantity, quality, and regulatory framework, from about 15000 basins from all over the world that, once aggregated, formed a composite index defined as *overall water risk* (Reig et al., 2013). Similar gridded approach was used to calculate the *Global Water Security Index (GWSI)*, an index based on information about water availability, accessibility, quality and management, aggregated through spatial Multi Criteria Analysis (Gain et al., 2016). Other examples exist at basin level spatial resolution, such as the *Transboundary Waters Assessment Programme (TWAP)* project (UNEP-DHI and UNEP, 2016). The hydro-political tension component in TWAP is part of the overall *Governance indicator*. This is based on three sub indicators: 1) *Legal Framework*, 2) *Enabling Environment*, and 3) *Hydro-Political Tensions.* The first is based on the rationale that governance of transboundary basins is driven by the existence of bi- or multi-lateral treaties regulating interactions between the countries. *Legal Framework* is based on the presence in the treaties of the following principles: (a) *“equitable and reasonable utilization*; (b) *not causing significant harm*; (c) *environmental protection*; (d) *cooperation and information exchange*; (e) *notification, consultation or negotiation*; (f) *consultation and peaceful settlement of disputes”* (quoted from UNEP-DHI and UNEP, 2016). The coverage of all the legal principles by the previous treaties, jointly with the ratification of the UN WC Convention and/or UNECE Water Convention by the countries involved, is considered a factor reducing risk. The *Enabling Environment* attains to the single countries’ capability of planning, regulating, managing, and governing water resources (UNEP-DHI and UNEP, 2016). The level of *Hydro-Political Tension* is obtained combining the institutional vulnerability with planned infrastructural development, where institutional vulnerability is higher in case the riparian countries did not specifically regulate in a formal treaty water allocation and management of flow variability, in case they did not agree on a conflict resolution mechanism, and in case the basin is not administrated by a RBO (UNEP-DHI and UNEP, 2016). The indicator was designed assigning a score to specific sub-indicators, then aggregated and ranked, following the methodology developed in existing literature (De Stefano et al., 2012, 2010b, 2010a). It is based on information derived from the water treaties database (International Freshwater Treaties Database - IFTD) (De Stefano et al., 2010b) created within the *Transboundary Freshwater Disputes Database* (TFDD) (Wolf et al., 2003). This work was then further developed in De Stefano et al. (2017). In this updated version, the current institutional resilience of the transboundary basins was calculated as a function of existing treaties and river basin managing institutions (RBO’s), similarly to the methodology used in the TWAP project (De Stefano et al., 2012; UNEP-DHI and UNEP, 2016). The hydro-political vulnerability of the basins was then quantified putting in relation with the institutional resilience destabilizing factors, such as planned infrastructural development, and the exacerbating factors, such as low income, climate driven water variability, reservoir depletion, armed internal or international conflicts, past water disputes through a multi-criteria analysis (De Stefano et al., 2017). The results were produced at basin level: thirty-six river basins were classified within the high and very high categories of hydro-political risk.

We propose a different, somewhat complementary, approach combining the information at transboundary basin level with local scale gridded data processed in an empirically based model designed to take into account linear and non-linear combinations between biophysical and socioeconomic stressors and international water interactions. In a second step, rather than assigning scores and aggregating sub-indicators in ranked relative risk categories, we used the model fit with past observations to construct a baseline and future projected scenarios. Similarly to other approaches described in this section, our index combines information at country level with gridded data, but, unlike previous approaches, our outcome variable is computed at gridded resolution. This makes the hereby proposed index spatially explicit and completely data driven.

## 2 Methodology and data

The empirically based analysis was designed upon concepts derived from political science and environmental economics, with a set of indicators selected covering information about: river basin freshwater availability; climate stress; human pressure on water resources; socioeconomic conditions, including institutional development and power imbalance; and topographic characteristics. A tool derived from machine learning, the Random Forests regression algorithm (Breiman, 2001), was used to estimate the relations between the indicators from each of the groups with observed water interactions. The relative impact of each time-varying and time-invariant indicator was in this way assessed and empirically estimated using the water related events database International River Basin Conflict and Cooperation – IRCC (Kalbhenn and Bernauer, 2012). The Random Forests regression model was trained based on historical information covering an eleven years period (1997–2007). Medium term mean (1997–2012) of the selected indicators at high spatial resolution (0.25 degrees) was then used to estimate the spatial distribution of the likelihood of experiencing hydro-political interactions (baseline scenario). Future scenarios of 2050 and 2100 were calculated by using the multi-model mean of the daily temperature and precipitation estimates from 5 GCM’s belonging to the Coupled Model Intercomparison Project Phase 5 (CMIP5) (Taylor et al., 2012), considering two different emission and radiative forcing scenarios, Representative Concentration Pathways (RCP) 4.5 and 8.5 (Meinshausen et al., 2011, 2009), for the 15 years period before the reference time (respectively, 2036–2050 and 2086–2100).

### 2.1 Data

Data about historical water interactions are the basis for hydro-political studies. Two main global dyadic databases of historical water related events are currently available: the Transboundary Freshwater Dispute Database (TFDD) International Water Event Database (IWED) developed by the Oregon State University with the Basins at Risk project (Wolf et al., 2003; Yoffe et al., 2003, 2004; and later updated in De Stefano et al., 2010b)^3^, providing information about international water basin interactions between 1948 and 2008; and the International River Cooperation and Conflict database (IRCC), reporting water related issues between 1997 and 2007 (Kalbhenn and Bernauer, 2012). Both databases are set up in the form of water related events at dyad-basin level. Each national territorial unit in a specific river basin is defined as a basin-country unit (BCU), each of the possible pairs of BCU’s in the same basin are classified as a dyad. Although the temporal coverage (11 years) is limited, the IRCC database was preferred in this analysis for the higher number of non-neutral interactions reported (4797 - IRCC vs 1985 - TFDD) (Kalbhenn and Bernauer, 2012), and for the data collection methodology coded from a homogeneous set of information (Bernauer and Böhmelt, 2014). The dyadic characterization of the database, with a geographical scale limited to bilateral country interactions for each transboundary basin, represents a limiting factor for a detailed spatial analysis of the biophysical and socioeconomic drivers determining the national and international water related issues. Moreover, due to the nature of the algorithms used for the creation of the database - mining water coded events from international news datasets - the event data are characterized by an uneven geographical distribution of the observations. More details and alternative water interactions databases are presented in the Annex A.

The hydro-meteorological information used in this analysis were derived from the highly spatially detailed climate data from the Multi-Source Weighted-Ensemble Precipitation (MSWEP) database (Beck et al., 2017). We calculated a precipitation anomaly indicator based only on variation in the temporal distribution of precipitation: the Standardized Precipitation Index (SPI) (McKee et al., 1993). This climate proxy, measuring rainfall anomalies, is widely used for drought quantification and monitoring (WMO, 2012), (details in Annex A). Temperature data were derived from the WATCH Forcing Data methodology applied to ERA-Interim (WFDEI) dataset (Weedon et al., 2014). Water availability was assessed using a modified version of the Falkenmark Water Stress Indicator (Falkenmark and Lannerstad, 2005), considering also the water resources flowing from upstream, calculated using the 0.1 degrees resolution LISFLOOD global hydrological model (De Roo et al., in preparation). River basin topographic data used for the analysis were mainly represented by the river flow accumulation, proxy for the upstream/downstream relations, and the share of national territory in the basin (Beck et al., 2014).

Gross Domestic Product (GDP) statistics were derived from Gleditsch (2002). The Governance indicator was calculated as mean value of the six indicators (voice and accountability; political stability and absence of violence; government effectiveness; regulatory quality; rule of law; control of corruption) of the Worldwide Governance Indicators (WGI) project (Kaufmann et al., 2010). Agriculture (share of GDP) and rural population (share of the total) were derived from the World Development Indicator database (World Bank, 2018.). Population dynamics were derived from the Gridded Population of the World (GPW, v4) database (CIESIN, 2015) downscaled by the EC Joint Research Centre (Freire and Pesaresi, 2015). Political and military importance of the countries was represented in the model through the Composite Index of National Capability (CINC) derived from the National Material Capabilities (NMC v5.0) database within the Correlates of War project (CoW) (Singer et al., 1972)^4^. CINC is calculated as a share of the world power as function of six variables, namely: total population, urban population, iron and steel production, military expenditure, military personnel, and primary energy consumption (Singer et al., 1972). The information about past bi- or multi-lateral water treaties were derived from the International Freshwater Treaty Database – IFTD (Oregon State University, Transboundary Freshwater Dispute Database TFDD)^5^ (De Stefano et al., 2012).

The climate projections data used in this study belong to the NASA Earth Exchange Global Daily Downscaled Projections (NASA NEX-GDDP) dataset downscaled (0.25 degrees) and bias corrected using the Bias-Correction Spatial Disaggregation (BCSD) methodology described in Thrasher et al. (2012). Due to computational constraints, we selected 5 out of the 21 climate models included in the NASA NEX-GDDP (details in Annex A), chosen on the basis of the structural differences among them, as described in Knutti et al. (2013).

Population density for the years 2050 and 2100 were estimated applying the country specific population growth rates estimated by the World Population Prospects of the UN/DESA (UN/DESA, 2017) to the population density used for the baseline scenarios (CIESIN, 2015; Freire and Pesaresi, 2015). Main statistics and variable description are summarized in Annex A (Table A1); further information about data sources could be found in Table A2.

### 2.2 Methodology: random forests regression

Different methodologies have been used in literature to analyze dyadic data. Most of them were not designed to capture the non-linear interactions. For this reason, in this work we propose a different approach applying the Random Forest (RF) regression algorithm (Breiman, 2001). RF is a Classification and Regression Tree (CART) based tool that involves an ensemble of regression trees. These are calculated on random subsets of data randomly split in base of specific features of each of the independent variables (Liaw and Wiener, 2002; Strobl et al., 2009; Welling et al., 2016). RF is based on the decision trees learning approach popular for non-linear multi-variate classification and regression (Breiman, 2001; Tin Kam Ho, 1998). In this study, we will refer to the RF regression, which is slightly different from the classification algorithm and is structured in four subsequent steps described below (RF algorithm logical steps, calibration, and validation procedures are summarized in Annex B).

*RF Model training*: the model was used to find the linear and non-linear relations between the dependent variable, a logarithmic transformation of the number of water interactions for each of the country-dyad/basin combinations observed for the 11 years (1997–2007) available data, and the 19 independent variables selected for the analysis (variable selection was performed optimizing the model performance as described in Annex B). Out of the 19 variables used in the final specification of the model (see Table A1 for details), the 2 representing the basin’s topography were time invariant, while the remaining 17, representing biophysical and socioeconomic factors were time varying (one is the time trend control). The interpretation of the modeling set up should be intended as the relation between a percent variation of the objective variable (the measure of the intensity of water issues), in response to the variation of absolute values of the independent variables. Since the relations, in the majority of the cases, are non-linear, by manipulating an independent variable, the variation of the objective variable could be positive or negative depending on the values of the remaining set of independent factors.

*Baseline*: the RF model set up in the previous step was used to construct a baseline (or reference) scenario of the likelihood of hydro-political issues at grid-cell level (each cell has dimensions 0.25 × 0.25 degrees, approximately 27 × 27 km at the equator). In order to reduce the bias derived by climate variability and possible temporary shocks in the specific independent variables, the baseline scenario was calculated by averaging the values of the independent variables for the period 1997–2012 at grid-cell level. Variables’ values at grid-cell level are cell specific (as for the 8 climatic variables^6^; population density^7^; and water availability^8^) or the same for all the cells of a country (as for all the socioeconomic variables^9^). The production of a baseline or reference scenario results in the possibility to map the spatial distribution of the likelihood of having water interactions, our index, at global level, upon present conditions of the factors determining water interactions.

*Projections*: using a procedure similar to the one described for the baseline, the model was used to map the variations on the objective variable as a response to four possible future climate and population scenarios. The future conditions are based on climate projections to the years 2050 and 2100 based on two different degrees of climate change (RCP 4.5 – moderate climate change scenario; and RCP 8.5 – severe climate change scenario). In order to reduce the bias derived from the specific climate modeling exercises, we averaged in a multi-model mean climate projections from 5 GCMs downscaled and bias corrected. Climate projections were combined with population growth scenarios at grid-cell resolution, calculated applying to the baseline population density (CIESIN, 2015; Freire and Pesaresi, 2015), country specific population growth rates for the years 2050 and 2100 (UN/DESA, 2017).

*Comparison* of the future and baseline scenarios to assess the change in the index caused by population and climate dynamics.

## 3 Modeling results

### 3.1 Random forest model results

**Fig. A3:**
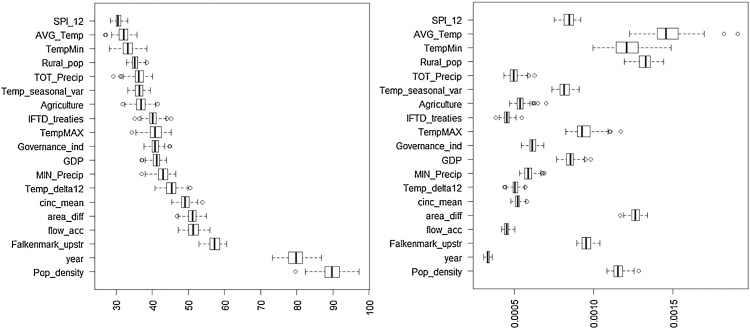
Permutation-based variable importance calculated running the model 100 times: importance expressed as percent increase of MSE (left) - Standard deviation of the permutation-based importance measure (right).

The RF model was trained using the entire set of observations (*N* = 11801). Each of the observations reports the logarithmic transformation of the number of hydro-political interactions for a specific dyad of countries (749 country dyadic combinations considered in the final panel) in a specific river basin (260 transboundary basins included) for a specific year (11 years). Of the final 11801 observations considered, 10062 reported no water interactions, while 1739 at least 1 interaction in the combination BCU/year^10^. The overall RF model was found to explain about 70% of the variation (pseudo R^2^, details available in Annex B). Variable importance estimates for the RF model highlighted that socioeconomic variables play the most important role. Population density was the variable that mostly influenced the capability of the model to capture the variation of the set of observations taken into account in this analysis. Time trend control resulted to be the second most important variable in capturing the variability of the data: this is likely due to the data collection algorithm of the hydro-political event dataset strongly influenced by the increasing of news availability in the period under consideration, coincident with internet development. The upstream/downstream dynamics (represented by the flow accumulation), jointly with territorial (area difference) and power imbalance (Composite Index of National Capability - CINC) follow the population dynamics. Per capita water availability (Falkenmark Index) was reported as the most important of the biophysical variables, while variables associated with precipitation and temperature follow in the mid-lower portion of the permutation-based variable importance ranking (Fig. A3).

**Fig. A4:**
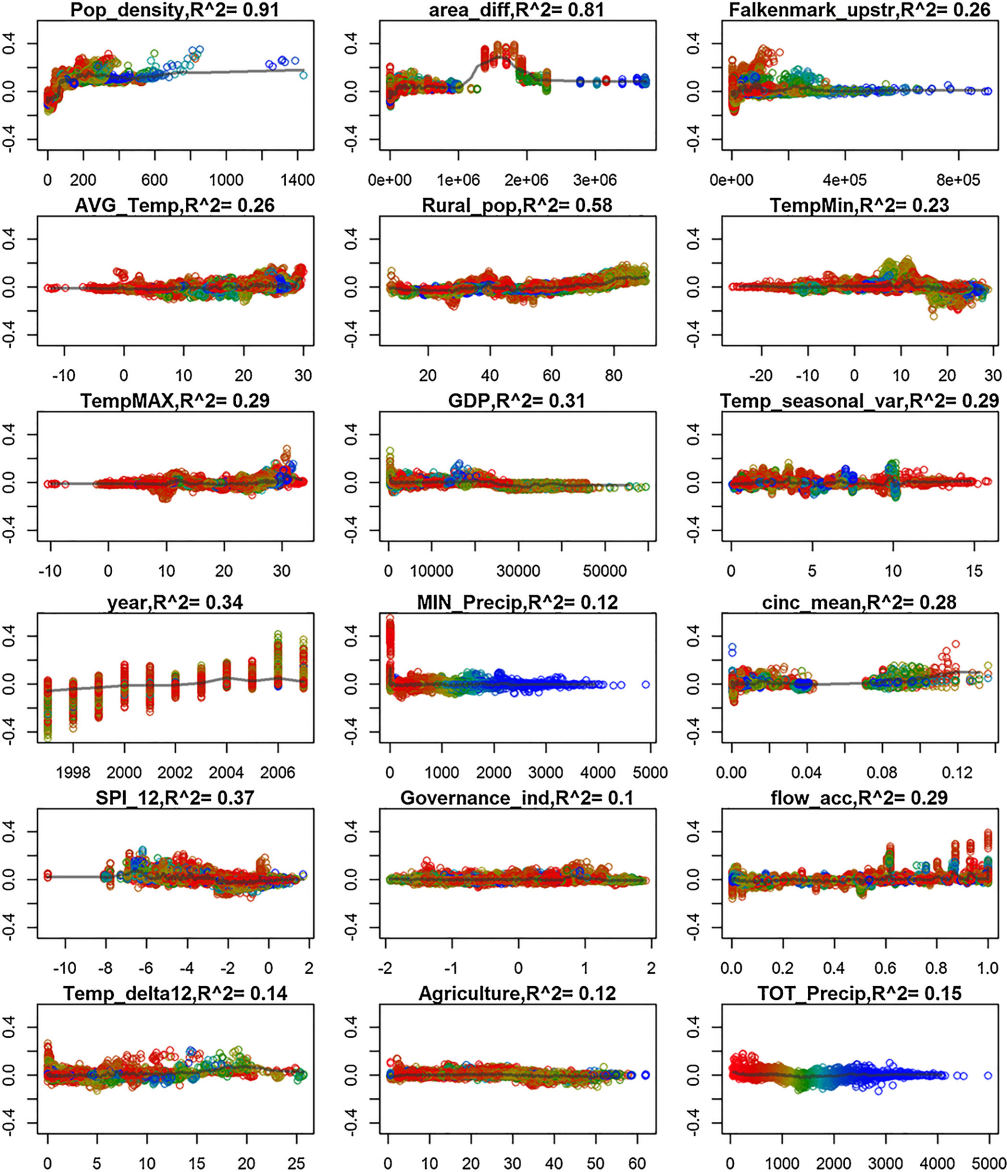
Partial Dependence plots. Y-axis indicates the cross validated contributions due to the explanatory variable (change in likelihood of hydro-political interactions due to changes in variable), X-axis indicates variable value. Colors indicate the mutual interactions between variables, while the coefficient of determination on top of the boxes indicates the goodness of fit of the trend line (Welling et al., 2016).

The performed analysis highlighted the non-linear nature of the relations between certain variables and their impact on the hydro-political interactions (further details in Annex B; partial dependence plots in Fig. A4). The model finds an increasing inverse U-shaped relation between population density and water interactions: sparsely populated areas were associated with a lower probability of having water issues; the likelihood increases till reaching its maximum at about 100 people km^−2^. Above this value the relation decreases remaining positive and leveling to zero for values above 400 people km^−2^. Almost opposite results are found for the Falkenmark Index, indicating per capita water availability including the amount of resources flowing from upstream: in the areas where the water availability is the lowest, increasing values are associated with a marginal decrease in the likelihood of water issues. The slightly negative relation is however non-linear: it is positive in areas where relatively more water is available and almost negligible in water abundant areas. Relative territorial supremacy on the basin (difference in the national territory in the shared watershed) was found to have an inverse U-shaped relation: the likelihood of water interactions appears to be very low among actors occupying similar territorial extensions of the shared river basin; similar conclusions could be drawn for countries occupying the majority of the basin territorial extensions, while hydro-political interactions are found to be more likely in the middle cases. Low to medium levels of national power (composite index of national capability) were found to be associated with higher likelihood of experiencing water interactions. Very upstream and very downstream countries are found to be more likely to get involved in water interactions. Rural and agricultural dependent economies and, in general, lower to middle income countries are more prone to experience water issues.

### 3.2 Model findings discussion

**Fig. A5:**
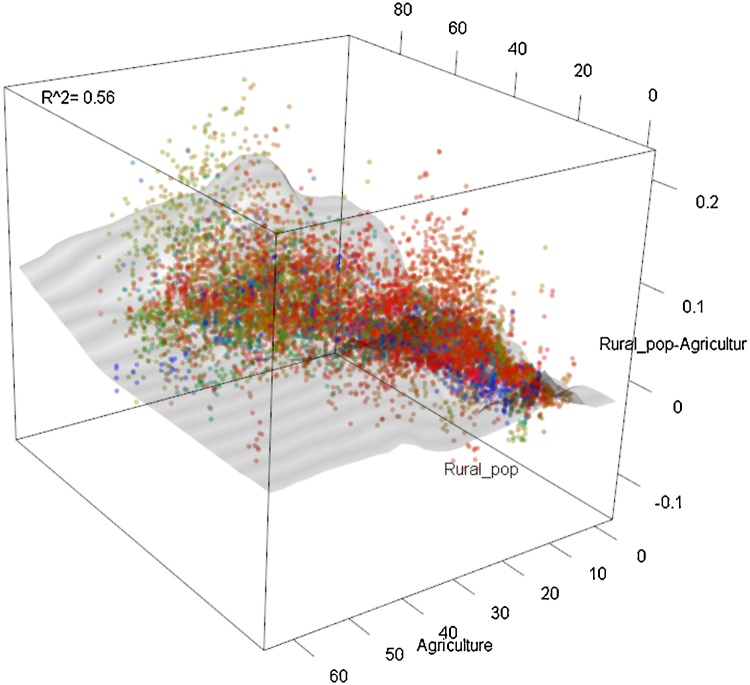
3-Dimensional partial dependency plot of the interactions between rural population and agricultural economic dependency and their combined impact on hydro-political interactions. Colors indicate the mutual interactions between variables, while the coefficient of determination on top of the boxes indicates the goodness of fit of the (grey) regression plane (Welling et al., 2016).

Socioeconomic and water demand side factors are found relatively more important in determining hydro-political interactions respect to supply side factors like shocks in precipitation or other climatic variables. Similar findings were highlighted in Böhmelt et al. (2014) where population pressure, agricultural productivity, and in general economic development were identified as important determinants for the formation of water disputes, mitigated into cooperative interactions in case of solid institutions and stable political conditions. Population dynamics were found important drivers in other studies. Brochmann and Gleditsch (2012), among others, found that countries characterized by very large or very small population are more likely to get involved in conflicts over water. In our case, population density is a proxy of human pressure over water resources, but population is also linked with the power of a nation and its economic and socio-political capabilities. Very low densely populated areas were found to be less likely to experience water interactions, but in case of rural communities (>50% of the population living in rural areas) extremely dependent on agricultural productivity (>30% of the GDP) for their economic development, the combination of the three factors was found likely to experience water issues (a 3-D dependence plot available in Fig. A5).

Increasing population density, by increasing human pressure on a limited set of resources, was found likely to increase the probability of experiencing water related issues, but this relation was found to be not linear. This could be explained considering the role of hydraulic infrastructures in mitigating water stress in densely populated areas (McDonald et al., 2014), and the extreme consequences when the capacity of the water infrastructures is no longer sufficient to cope with climatic variability and population growth. Similarly, in Dinar et al (2011) increasing human pressure on water resources, determining water scarcity, was found to have an inverse U-shaped relation with cooperative hydro-political interactions, while extreme cases were more associated with tensions. The inverse U-shaped relation between per capita water availability and likelihood of hydro-political interactions confirms also the conclusions of Dinar et al (2010), that found cooperative water interactions more likely in situation of average water availability. Territorial and power imbalance were found significant drivers of hydro-political interactions in the main literature available (Brochmann and Gleditsch, 2012; Gleditsch et al., 2006; Zawahri and Mitchell, 2011). This study’s findings about upstream/downstream dynamics confirm the accurate study performed by Munia et al. (2016) quantifying the increasing water stress in the downstream part of the basins due to upstream uses, and its connection with increasing water tensions. Our results found an increasing trend of water related interactions over time. On the one hand, the institutional development brought an increasing collaboration over water related international issues (De Stefano et al., 2012; Dinar et al., 2015; Kalbhenn and Bernauer, 2012; Wolf, 2009). On the other hand, the trend is (at least partially) explained by the increasing coverage of the international press industry of the local news about water issues. The way water event datasets were developed, in fact, is strongly influenced by the publication of news in the main western languages: this sector has been radically influenced by the digital revolution. As noted in De Stefano et al. (2010b), the scarce representation of some areas of the world in the water related events datasets is mainly due to the fact that the search was performed analyzing international and local news in English. This methodology proved to be rather unsuccessful in capturing information published in local languages or news from area not completely covered, such as war zones or politically or technologically isolated countries. For this reason, data about historical water related events represent the main limitation of the studies in this specific field.

## 4 Model application to calculate the likelihood of hydro-political interactions under current and upcoming conditions

**Fig. 1:**
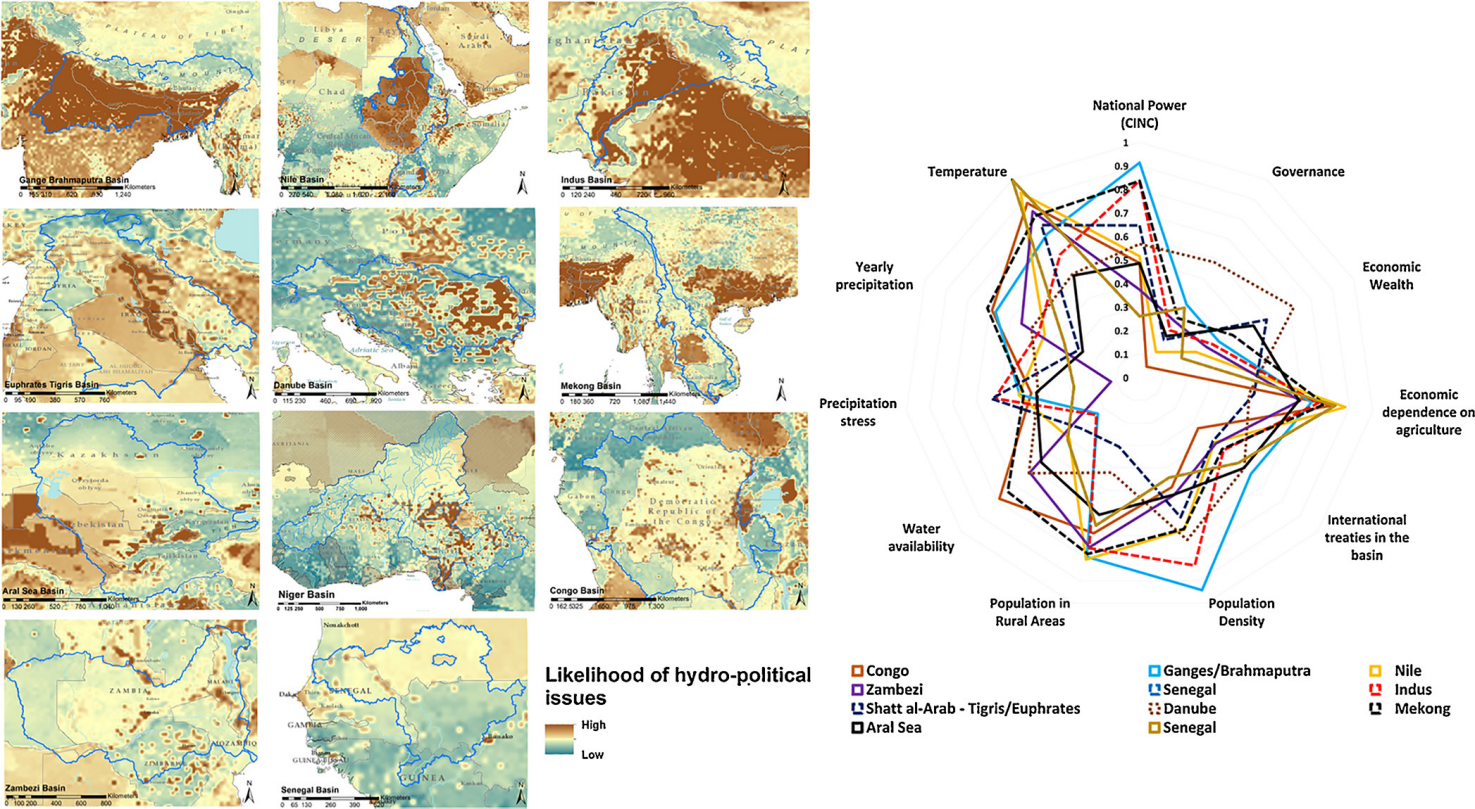
Likelihood of the occurrence of hydro-political interactions in the main transboundary river basins (from the top-left [normalized likelihood of hydro-political issues, min = 0 and max = 1]: Ganges-Brahmaputra [1.000], Nile [0.761], Indus [0.675], Euphrates-Tigris [0.592], Danube [0.499], Mekong [0.492], Aral Sea [0.455], Niger [0.447], Congo [0.432], Zambezi [0.431], Senegal [0.372]). In the radar chart the normalized score of the main factors determining the likelihood in the specific river basins. Not all the variables explicitly used for the model are represented in the radar chart: the non-included factors, however, are derived from the climatic variables displayed.

One of the main objectives of this study was to draw a spatially explicit data driven index aimed to help the policy makers in monitoring the dynamics of the factors identified as influential in determining water related issues, and in identifying the areas where cooperation over water is more needed to timely address criticalities that could eventually lead to water disputes. In order to achieve this objective, we calculated the medium-term mean (1997–2012, when available) of the selected indicators at the highest spatial resolution allowed by data availability (0.25 degrees), and we used the estimated RF model to draw the spatial distribution of the likelihood of hydro-political interactions. Not all the variables were available at sub-country resolution, in particular: 10 variables were available at grid-cell level; 5 at country level; 3 at country/basin level (more details in Table A1). The spatial distribution of the index within the country borders is therefore driven by climatic, population, and water availability drivers: an unavoidable simplification caused by the limited availability of data at sub-country and gridded level, partially compensated by the fact that the spatial distribution of some variables, as for the national capability (CINC), can be considered fairly homogeneous at intrastate level. A high likelihood of hydro-political interactions identifies the areas where water issues are more probable to raise. Although this index does not give information about the degree of cooperation or conflict associated with the specific interaction, it identifies the areas of possible hydro-political risk that would be best addressed through a cooperative action (Fig. 1). The index was calculated at pixel level, the values attributed to each specific basin is the average of all the pixels within its boundaries. To ensure the comparability of the different variables and indicators, the corresponding values were normalized across the transboundary basins in a 0–1 range through a simple min-max normalization procedure. High values of the likelihood of hydro-political interactions are linked with a larger water stress, due to lack of water supply and/or human pressure in a more vulnerable institutional and socioeconomic context. The spatial distribution of the index highlights the areas where it could be more likely to experience issues related to water resources. High likelihood of water related issues could be determined by potential water scarcity in densely populated areas, as in the case of the Nile Delta, one of the basins that reach an high average value of the index (score 0.761). Socioeconomic, political conditions and distribution of water resources determine the differences in the index for the Upper Nile. A combination of low governance, high population density, physical water stress, and almost complete economic dependency on agricultural activities, shaped the distribution of the index in the Ganges-Brahmaputra (highest in our ranking, score 1.000), and Indus basins (score 0.675). A different climatic area, more pronounced precipitation stress, with a lower population density and lower economic dependency on agricultural production characterized the results for the Euphrates-Tigris river basin (score 0.592). Population density, high economic dependence on agriculture, and human pressure on water resources determine the distribution of the index on the lower Niger (score 0.447), in particular within the borders of Burkina Faso and Nigeria. Population distribution and socioeconomic conditions shape the index in the Congo basin (score 0.432), while a relatively good governance level characterizes the Zambezi river basin, with hotspots in the most populated areas, and increasing values towards the outlet of the basin (overall score 0.431). Human pressure and relatively heterogeneous socioeconomic conditions determine the need for water cooperation in the Mekong basin (score 0.492). Despite the evident progresses made after the EU integration, our results highlight high likelihood of water related issues in specific portions of the Danube basin (score 0.499), especially in the eastern and southern parts, where there is still need to consolidate institutional development and the economic dependency on agriculture still remains relevant. A complete list of the results for the transboundary river basin is available in the Annex D (Table A3).

**Fig. 2:**
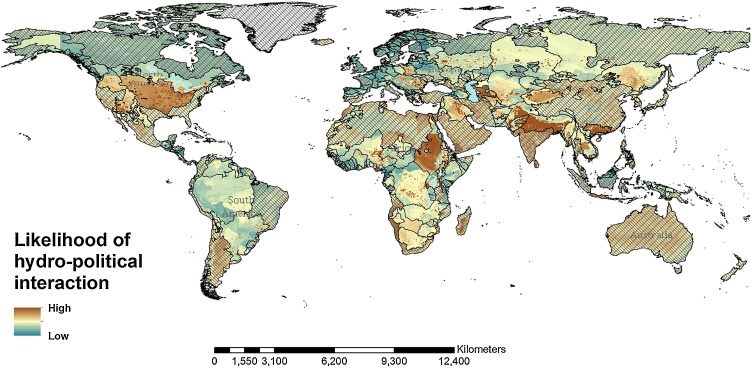
Global distribution of the current likelihood of hydro-political issues among the main transboundary basins (transboundary basin borders in black, non-transboundary areas shaded).

**Fig. A7:**
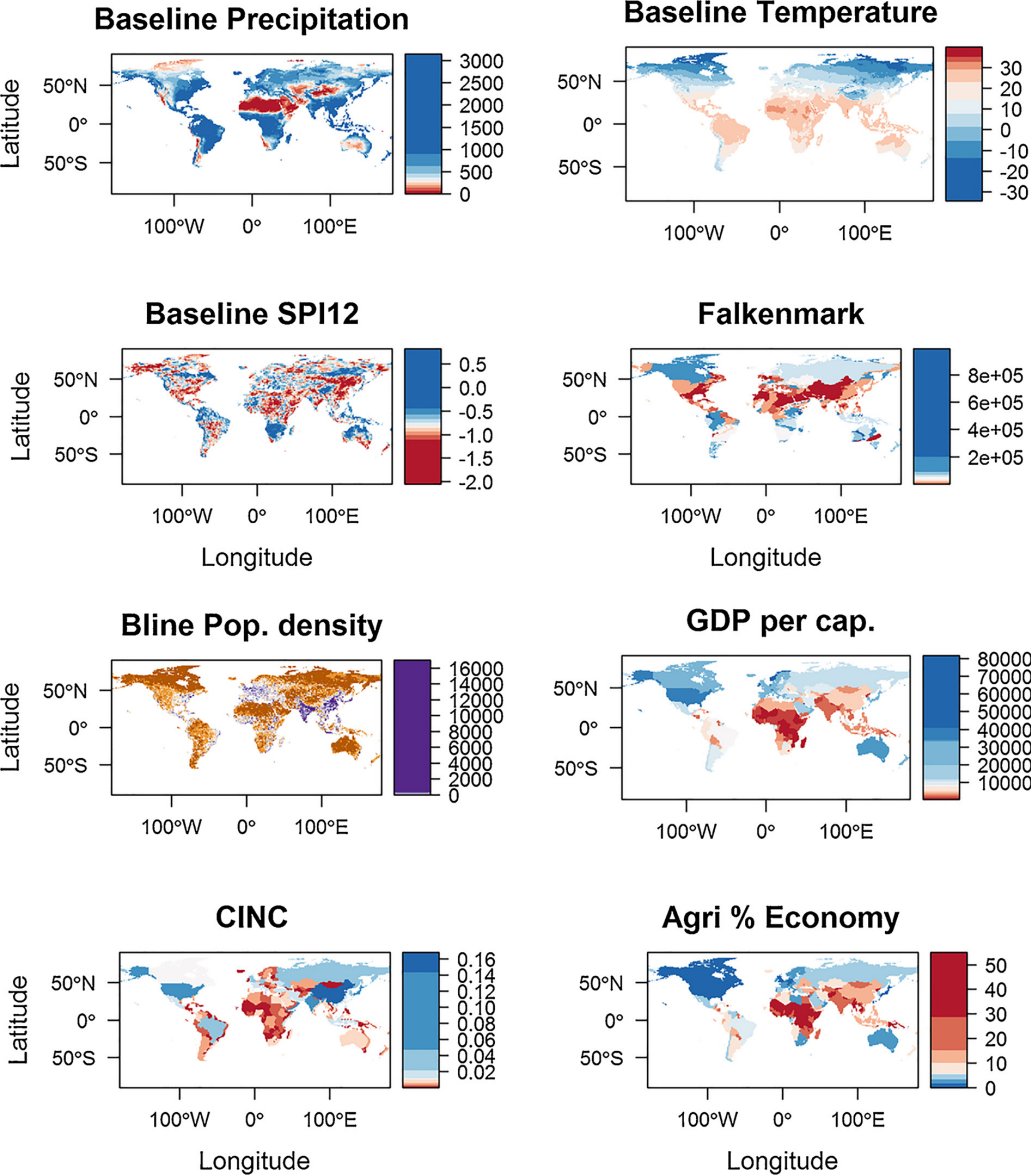
Input Data Baseline Scenario.

Some of the areas highlighted in the results shown above (and in Table A3) are well known hotspots for hydro-political issues. Other areas are scenarios of national or international political tensions not directly related with water. Although, given the different nature of our study focusing on water interactions as a measure of the magnitude of water issues, a direct comparison with previous studies aiming to identify basins at risk of future water tensions is not possible, the results of the different approaches are aligned. De Stefano et al. (2017) compared the *basins at risk* identified using their approach with the ones highlighted in the two previous assessments (Bernauer and Böhmelt, 2014; Wolf et al., 2003). Of the 12 basins found to be more likely to experience water issues in this study (Table A3), 10 are identified as *basin at risk* in previous analyses, namely: Ganges/Brahmaputra (Wolf et al., 2003), Pearl/Bei Jiang (De Stefano et al., 2017), Nile (Wolf et al., 2003), Feni (or Fenney) (Bernauer and Böhmelt, 2014), Indus (Bernauer and Böhmelt, 2014; Wolf et al., 2003), Colorado (Bernauer and Böhmelt, 2014), Tarim (De Stefano et al., 2017), Shatt al-Arab - Tigris/Euphrates (Bernauer and Böhmelt, 2014; Wolf et al., 2003), Hari (Bernauer and Böhmelt, 2014), and Irrawaddy (De Stefano et al., 2017; Wolf et al., 2003). Therefore, the probability of observing hydro-political interactions is to some extent correlated with the hydro-political risk analyses conducted in previous studies identifying basins at risk. That supports the idea that the index proposed herein should be considered for systematic application in support to the assessment of the SDG 6, in particular for what concerns the impacts of future potential biophysical or socio-environmental changes on the likelihood of hydro-political issues at global scale. The proposed index can also be used to assess interlinkages with other SDGs, and in particular SDG 16 on peace, justice and institutions. In order to achieve a global perspective, our analysis was extended also outside the borders of the international river basins initially included in the analytical framework (Fig. 2 and Fig. A7 in the Annex). The results outside the boundaries of the international river basins and in the portions of them not or poorly represented in the database of hydro-political events used to fit the RF model, might be affected by certain degrees of error and, that for, should to be considered purely indicative.

**Fig. 3:**
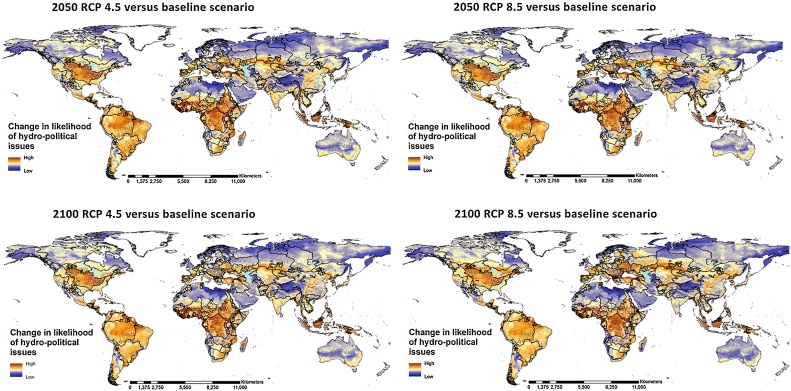
Change in the likelihood of hydro-political issues considering the four future climate change and population scenarios respect to the baseline presented in Fig. 2.

**Fig. A8:**
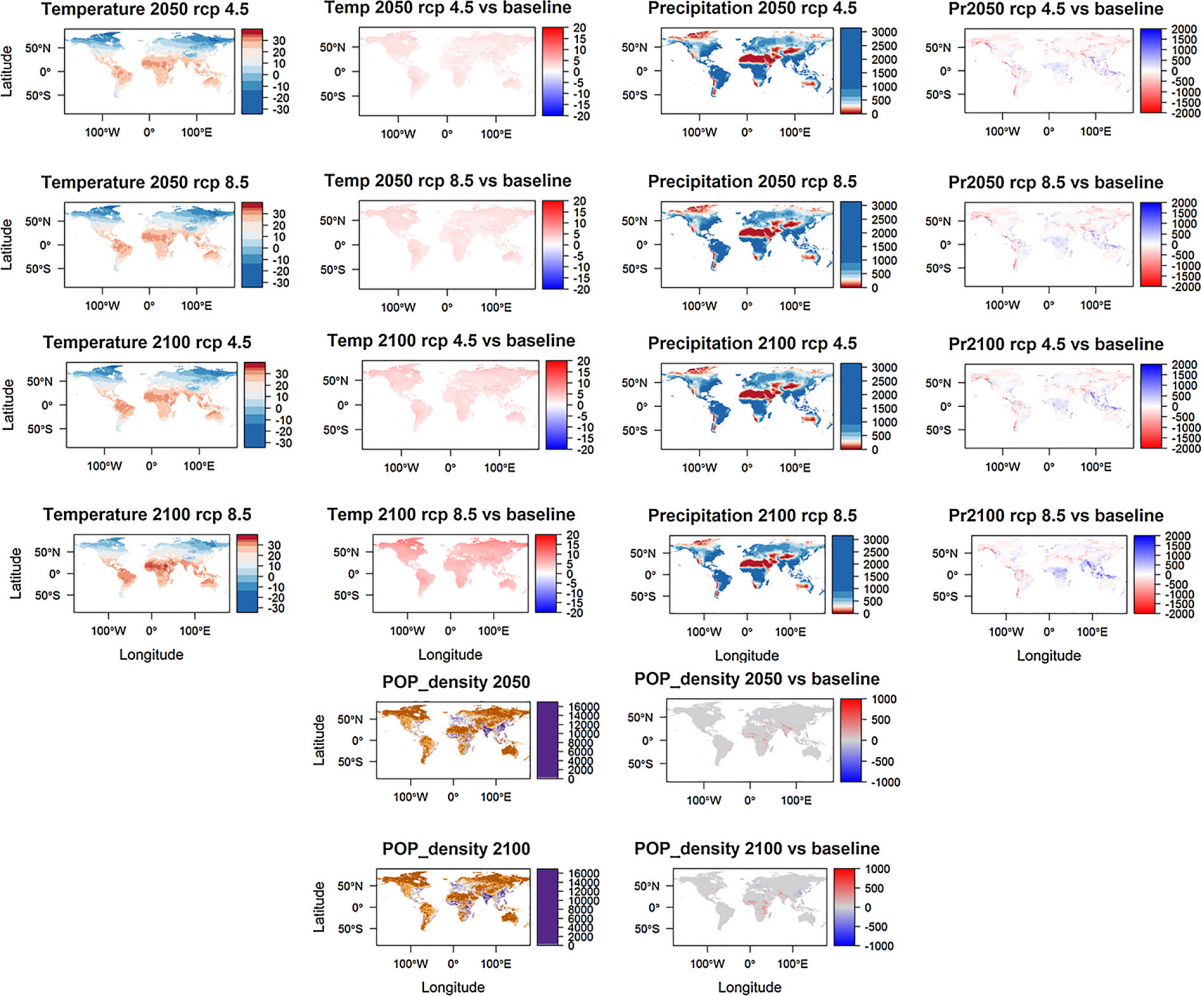
Input data projected scenarios.

**Fig. A9:**
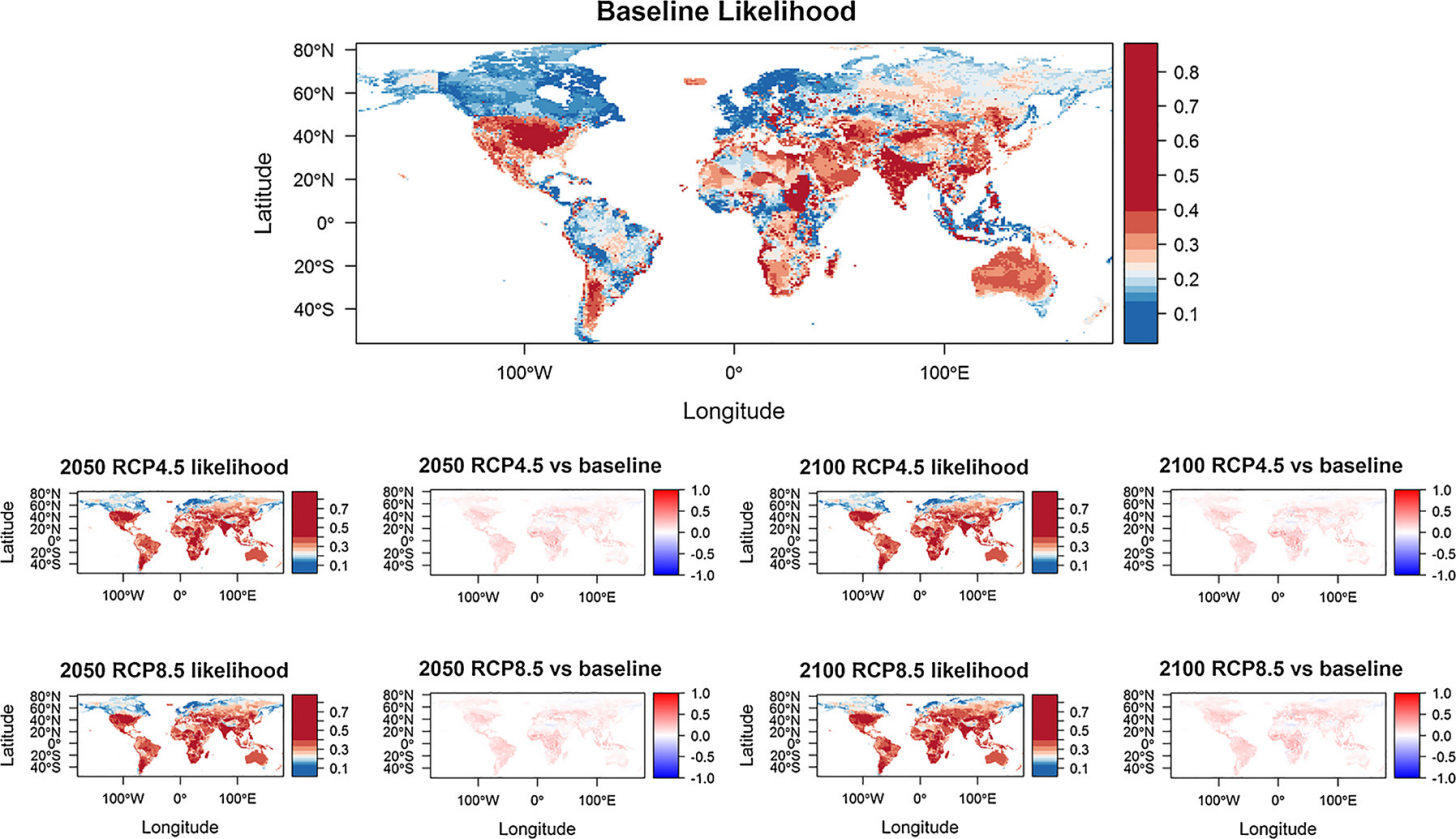
Baseline and projected scenarios results.

The evolution of the index under future climate and population scenarios was estimated for the years 2050 and 2100 considering changes in population density, by applying UN/DESA population growth estimates to the 2015 data, and climate conditions, considering the multi-model-mean of the projected precipitation and temperature for the periods 2036–2050 and 2086–2100 (Fig. 3 – Additional details in the Annex – Fig. A8, Fig. A9). As mentioned above, population density is among the top drivers determining the likelihood of hydro-political interactions, while, conversely, climate factors are relatively less important in terms of magnitude, but more relevant in terms of impacted area extent. The reason for choosing the combination of climate and population dynamics as driver for change is motivated mainly by data availability. When alternative scenarios of other important variables and relevant dynamics, as for instance the institutional development, will be available, these could be taken into consideration as well.

Changes in population density are expected to exacerbate the anthropogenic pressure on water resources, the availability of which is strongly impacted by changes in climate. The combination of these two factors is estimated to impact negatively on the overall hydro-political risk. The likelihood of water related issues is expected to increase globally, with gridded values averaging between +36.4% in the case of moderate climate change (RCP 4.5) and +37.1% in the case of the more pessimistic climate scenario (RCP 8.5) for the year 2050, and respectively between +39.3 and +46.8% for the year 2100. Aggregating the results for the main transboundary river basins, excluding the areas of the globe not falling in transboundary basins, the likelihood of experiencing hydro-political interactions was calculated to increase on average between 74.9% (2050 RCP 4.5) to 95.3% (2100 RCP 8.5), especially in sub-Saharan Africa, South America, Southern North America, Southern and Eastern Europe, Central and Southern Asia. Table 1 presents the main statistics for the global projections, and the results for the transboundary basins most represented in the original IRCC database that were found to be likely of experiencing more hydro-political interactions in the future. The convergence of the increasing trends in population density and temperature, jointly with decreasing precipitation is the combination that most influences the future increasing hydro-political risk, as for instance in the case of Southern Europe, Central Asia, and Middle East (Fig. A8, Fig. A9). Increasing population and temperature were found to be dominant respect to increasing precipitation, as in the case of some tropical areas in sub-Saharan Africa and South-East Asia, in some cases due to the seasonal distribution of the rainfall. Harsher climate conditions were found to offset the benefits derived by decreasing population density, as in the case of North-Eastern China in the second half of the 21st Century.

**Table 1:** Summary of estimated change of the likelihood of experiencing hydro-political interactions under four future projected scenarios. Data are presented aggregated per geographic areas or river basins. Values are presented as average (minimum and maximum variation). A more comprehensive table is presented in the Annex (Table A3).

	2050 RCP 4.5		2050 RCP 8.5		2100 RCP 4.5		2100 RCP 8.5	
	Avg % change (Min / Max)	St. Dev	Avg % change (Min / Max)	St. Dev	Avg % change (Min / Max)	St. Dev	Avg % change (Min / Max)	St. Dev
Globe	36.4 (-72/+5944)	56.3	37.1 (-71.5/+5861)	56.9	39.3 (-76.5/+5120)	60.9	46.8 (-69.9/+5235)	67.5
Transboundary basins (all)	74.9 (-61/+5944)	66.7	76.2 (-61/+5861)	97.3	80.7 (-66/+5120)	72.1	95.3 (-57/+5235)	79.4
Lake Chad	77.2 (+12.8/+439)	48	76.7 (+11.7/+439)	48.3	85.7 (+10.9/+567)	67.3	78.4 (-4.2/+557)	64.4
Congo	70.9 (-13.7/+547)	71.6	71 (-12/+546)	70.9	78.1 (-4.6/+601)	75.7	83.3 (-3.6/+514)	72.7
Niger	64.1 (-2.3/+346)	50.6	62.3 (-3.6/+333)	47.9	76 (-3/+378)	68.8	66 (-8.1/+339)	59.5
Nile	43.3 (-35.5/+599)	70.5	43.1 (-35.5/+607)	69.9	45.2 (-30.1/+697)	85	42.4 (-32.5/+734)	84.2
Zambezi	38.9 (-6.4/+321)	34.2	38.5 (-5.8/+312)	33.8	48.4 (-7.7/+418)	47.4	47.1 (-10.2/+342)	43.7
Senegal	36.7 (-5.7/+234)	48.7	36.4 (-5.9/+235)	48.5	45.4 (-5.8/+265)	59.4	41 (-5.1/+247)	51.7
Aral Sea	33.2 (-11.2/+249)	30.1	34.2 (-12.9/+252)	31.1	35.6 (-12.1/+259)	32.1	41.9 (-16.4/+292)	37.4
Euphrates-Tigris	23.1 (-2.5/+349)	46.4	23.5 (-2.3/+364)	48.4	26.5 (-3.5/+446)	50.4	32.5 (-5.3/+563)	59.1
Danube	24.2 (-55/+510)	82.7	26 (-55.5/+518)	84.7	19.2 (-66.1/+555)	88.3	34.7 (-52.3/+651)	110
Indus	12.3 (-27/+169)	26.5	12.5 (-32.5/+168)	26.8	15.3 (-34.9/+224)	30.3	19.1 (-32.3/+262)	35

Only a handful of transboundary basins are expected to benefit or not being impacted by the global climatic and population changes: one in Central Asia, Chuy Basin (from -8% 2050 RCP 4.5 to -37% 2100 RCP 4.5); two in the North of the Scandinavian peninsula: Tuloma Basin (between Russia and Finland, from -3% 2050 both RCP’s to +3% 2100 RCP 8.5), and Näätämö basin (at the border between Finland and Norway, -3.2% 2050 RCP 8.5 to +1% 2100 RCP 8.5); and two in Ireland: Bann Basin (-13.4% 2100 RCP 4.5 to +2% 2100 RCP 8.5), and Flurry Basin (-17.4% 2100 RCP 4.5 to -1.6% 2100 RCP 8.5). All these basins are characterized by low population density and, the ones in the northern latitudes, abundant water availability. A detailed list of the projected population and climatic variables, and the estimated results in terms of hydro-political risks are available in the Annex (Figs. A8, A9, and Table A3, respectively).

The increasing pressure that future climate and population dynamics are expected to pose upon the already problematic basins, especially in the Sahelian and Sub-Saharan Africa, Central, South and South-Eastern Asia, should be carefully monitored in order to avoid eventual hydro-political turmoil. In particular, the institutional and governance capacity of the national and supranational institutions (RBO’s) should be enhanced in order to minimize the vulnerability of the specific biophysical and socioeconomic basin-systems to the increasing pressure. This aspect could significantly increase the capability of the river systems to deal with the increasing magnitude of change.

## 5 Conclusion

In this paper, we presented an innovative analysis of the past hydro-political issues in international river basins and their determinants through the application of the Random Forest regression algorithm. Our analysis had two main goals: highlighting the factors that are more relevant in determining the hydro-political interactions, capturing also the non-linear relations between the main drivers; and producing a tool able to map and monitor the evolution of the hydro-political risk over space and time, under specific socioeconomic and biophysical scenarios. We did that by designing an empirically estimated, data-driven, and spatially explicit global index of the magnitude of hydro-political issues. The factors that were found to be more relevant in determining hydro-political interactions were mainly represented by, respectively: population density, water availability (quantified through the Falkenmark index), upstream/downstream dynamics (represented by the flow accumulation), with territorial (area difference) and power imbalance (Composite Index of National Capability – CINC), and climatic conditions. Current climatic and socioeconomic conditions were used to design a baseline scenario of the distribution of the likelihood of hydro-political interactions. This output allows to map the spatial distribution of the areas within the basins where water management issues are more likely to rise under current conditions. Among the basins found to be more likely to experience water issues in this study, some were already identified as basin at risk in previous analyses, namely: Ganges/Brahmaputra, Pearl/Bei Jiang, Nile, Feni (or Fenney), Indus, Colorado, Tarim, Shatt al-Arab - Tigris/Euphrates, Hari, and Irrawaddy. The hereby proposed index adds the possibility to identify the most critical areas within the basin boundaries. The baseline scenario was then compared to four distinct climate and population density projections, designed by combining the most updated bias corrected and spatially detailed climate and the most recent estimates of the future population changes. The results of this work allow the identification of the areas where water interactions are more likely to arise under present and upcoming conditions, and cooperation over water should be pursued to avoid possible hydro-political tensions. Future demographic and climatic conditions are expected to heavily increase the probability of experiencing water management issues in already stressed basins, such as the Nile, the Indus, the Colorado, the Feni, the Irrawaddy, the Orange, and the Okavango.

One of the characteristics of the analysis presented is that we chose not to make a distinction between past episodes of cooperation and dispute over water, using them collectively as water interactions, a measure of the magnitude of the associated water issue. This was motivated by the fact that water disputes had virtually never ended in violent conflicts, at least in the most recent historical eras, and by the consideration that the classification of positive (cooperative) and negative (conflictive) interactions in the event databases has often been arbitrary and ambiguous. Our focus was then more oriented towards understanding the preconditions increasing the likelihood of experiencing hydro-political interactions due to emerging water management issues. More than being exhaustive, our approach tends to boost the interest in the hydro-political field of study, by offering a new perspective through the application of a methodology that had never been considered before in this kind of analyses, dealing with aspects that are different by the only institutional resilience, and by exploring the possibility of creating a spatially explicit interactive tool able to assist stakeholders and policy makers in dealing with water related issues in different socioeconomic and climatic contexts through the analysis of *what-if* scenarios. Future studies could further develop the instrument by integrating updated socioeconomic, biophysical, and demographic projections.

The difficulties and the limitations encountered in this process were multiple. Beside the logical constraints that every global analysis has, as the other studies in this field, this work is affected by limitations in data availability. Water events database are extremely hard and expensive to collect and to manage. Data collection is mostly conducted through the application of mining algorithms operating in the news databases available only in the most widely spoken western languages. For this reason, the available datasets are necessarily biased and incomplete. Their temporal coverage is very limited, only eleven years in our case, and the sub-national geographic characterizations of the specific water related events is, in the majority of the cases, not considered. These particular factors make very difficult to apply the existing datasets for the development of spatially explicit interactive decision making tools.

As stated above, the index presented in this paper could be applied for the Agenda 2030 monitoring activities and in particular for Target 6.5 – *Water Resources Management*, where the only indicator regarding hydro-political dynamics used is the 6.5.2 *Proportion of transboundary basin area with an operational arrangement for water cooperation*. This is an indicator capturing mainly the institutional resilience in transboundary basins, with no consideration for the other determining factors specifically analyzed in this study. Therefore, the use of the proposed index could provide a substantial contribution to move from the mere recording of facts, to the understanding of phenomena the mechanisms behind them, which are prerequisites for identification of effective sustainability policies.

As noted already in previous global analyses (Bernauer and Böhmelt, 2014; De Stefano et al., 2017; Yoffe et al., 2003), the results of this study should be intended to be an indicator of the areas that might require closer investigation under present and possible upcoming scenarios. We recommend to further explore the development of this analysis in regional or sub-regional contexts where more detailed data is available. Future research will be focused in specific transnational river basins in developing countries where potential water stress exacerbated by climate change and variability, rapid population growth, and unsustainable development could be further destabilizing factors for the already tumultuous political context.

## Author attribution

F. Farinosi, G. Bidoglio, A. Reynaud, and C. Carmona-Moreno designed the study; F. Farinosi and G. Ceccherini developed the modeling framework; F. Farinosi processed data, coded the methodology, and performed the analysis; F. Farinosi, C. Giupponi, A. Reynaud, A. De Roo, G. Bidoglio, and C. Carmona-Moreno discussed the results; F. Farinosi with comments from the co-authors wrote the manuscript.

## Conflict of interest

The authors declare no conflict of interest.

## Funding

Arnaud Reynaud gratefully acknowledges the financial support of the Research Chair "Finance Durable et Investissement Responsable" and the Research Chair Amundi.

## Figures and Tables

**Table A1:** Descriptive statistics of the data used in the analysis.

Group / Sub-group Name	Description	Abbreviation	*Unit*	Mean	SD	Min	Max	Spatial resolution	Time varying?
								
**Biophysical**									
Falkenmark index	Per capita water available including upstream flow	*Falkenmark_upst*	* m^3^ year^−1^*	52,816	80,549	146	904,414	Grid-cell	Y
Precipitation	Total precipitation	*TOT_Precip*	*mm year^−1^*	1,028	596	31	4,971	Grid-cell	Y
	Minimum precipitation	*MIN_Precip*	*mm year^−1^*	764	607	0	4,910	Grid-cell	Y
	Standard Precipitation Index	*SPI_12*	–	−2.5	1.66	−10.8	1.7	Grid-cell	Y
Temperature	Average temperature	*AVG_Temp*	*°C*	16.36	8.86	−12.81	30.02	Grid-cell	Y
	Maximum temperature	*TempMAX*	*°C*	20.01	8.44	−10.51	33.68	Grid-cell	Y
	Minimum Temperature	*TempMin*	*°C*	9.95	10.68	−26.39	28.79	Grid-cell	Y
	Temperature variation	*Temp_delta*	*°C*	3.21	3.71	0	25.69	Grid-cell	Y
	Seasonal variability	*Temp_seasonal_var*	*Stand. dev.*	2.93	2.51	0.03	15.85	Grid-cell	Y
**Socioeconomic**									
Population	Population density	*Pop_density*	*People sqkm^−1^*	67.62	89.30	0	1,433	Grid-cell	Y
	Share of rural population	*Rural_pop*	*%*	49.42	19.52			Country	Y
GDP	Per capita Gross Domestic Product	*GDP*	*2005 USD*	8,659	10,013	313	59,384	Country	Y
Agricultural dependency of the economy	Agricultural share of the GDP	*Agriculture*	*%*	17.60	12.92	0	61.96	Country	Y
Institutional development and quality	Worldwide Governance Indicators (WGI)	*Governance_ind*	*Normalized value (range: -2.5/+2.5)*	−0.274	0.83	−1.939	1.910	Country	Y
Power imbalance	Composite Index of National Capability	*cinc_mean*	*Normalized value (range: 0/1)*	0.010	0.021	0	0.136	Country	Y
Previous collaboration	Number of bi-or multi-lateral water treaties	*IFTD_treaties*	*number*	17.84	15.61	1	95	Country/ basin	Y
**Topography**									
Flow accumulation	% of the flow accumulated in the country	*flow_acc*	*%*	0.436	0.31	0	1	Country/ basin	N
Territorial imbalance	Difference of national territory in the basin	*area_diff*	*‘000 km^2^*	190.8	455.4	0	3,739	Country/ basin	N
**Others**									
Time trend	Time trend variable	*year*	–	–	–	–	–		Y
								
**Projected Scenarios - Climate**									
Precipitation	Total precipitation 2050 rcp 4.5	*TOT_Precip_2050_45*	*mm year^−1^*	723	691	0	7,359	Grid-cell	Y
	Total precipitation 2050 rcp 8.5	*TOT_Precip_2050_85*	*mm year^−1^*	730	695	0	7,566	Grid-cell	Y
	Total precipitation 2100 rcp 4.5	*TOT_Precip_2100_45*	*mm year^−1^*	745	715	0	7,780	Grid-cell	Y
	Total precipitation 2100 rcp 8.5	*TOT_Precip_2100_85*	*mm year^−1^*	794	765	0	8,879	Grid-cell	Y
	Minimum precipitation 2050 rcp 4.5	*MIN_Precip_2050_45*	*mm year^−1^*	9	17	0	252	Grid-cell	Y
	Minimum precipitation 2050 rcp 8.5	*MIN_Precip_2050_85*	*mm year^−1^*	9	17	0	274	Grid-cell	Y
	Minimum precipitation 2100 rcp 4.5	*MIN_Precip_2100_45*	*mm year^−1^*	9	18	0	278	Grid-cell	Y
	Minimum precipitation 2100 rcp 8.5	*MIN_Precip_2100_85*	*mm year^−1^*	9	19	0	351	Grid-cell	Y
Temperature	Average temperature 2050 rcp 4.5	*AVG_Temp_2050_45*	*°C*	11.86	13.70	−25.31	32.67	Grid-cell	Y
	Average temperature 2050 rcp 8.5	*AVG_Temp_2050_85*	*°C*	12.22	13.69	−25.16	33.01	Grid-cell	Y
	Average temperature 2100 rcp 4.5	*AVG_Temp_2100_45*	*°C*	12.90	13.49	−23.56	33.75	Grid-cell	Y
	Average temperature 2100 rcp 8.5	*AVG_Temp_2100_85*	*°C*	15.76	12.86	−18.57	36.03	Grid-cell	Y
	Maximum temperature 2050 rcp 4.5	*TempMAX_2050_45*	*°C*	20.01	8.44	−10.51	33.68	Grid-cell	Y
	Maximum temperature 2050 rcp 8.5	*TempMAX_2050_85*	*°C*	29.72	8.96	−5.61	48.56	Grid-cell	Y
	Maximum temperature 2100 rcp 4.5	*TempMAX_2100_45*	*°C*	30.72	8.97	−4.99	49.86	Grid-cell	Y
	Maximum temperature 2100 rcp 8.5	*TempMAX_2100_85*	*°C*	33.54	8.89	−2.39	53.37	Grid-cell	Y
	Minimum temperature 2050 rcp 4.5	*TempMin_2050_45*	*°C*	−5.86	19.44	−53.79	27.47	Grid-cell	Y
	Minimum temperature 2050 rcp 8.5	*TempMin_2050_85*	*°C*	−5.46	19.33	−53.53	27.76	Grid-cell	Y
	Minimum temperature 2100 rcp 4.5	*TempMin_2100_45*	*°C*	−4.59	18.97	−52.00	28.16	Grid-cell	Y
	Minimum temperature 2100 rcp 8.5	*TempMin_2100_85*	*°C*	−1.28	17.65	−47.29	29.69	Grid-cell	Y
	Temperature variation 2050 rcp 4.5	*Temp_delta_2050_45*	*°C*	11.29	2.91	0.48	21.23	Grid-cell	Y
	Temperature variation 2050 rcp 8.5	*Temp_delta_2050_85*	*°C*	11.23	2.91	0.46	21.38	Grid-cell	Y
	Temperature variation 2100 rcp 4.5	*Temp_delta_2100_45*	*°C*	11.27	2.97	0.47	21.51	Grid-cell	Y
	Temperature variation 2100 rcp 8.5	*Temp_delta_2100_85*	*°C*	11.06	3.04	.47	21.74	Grid-cell	Y
	Seasonal variability 2050 rcp 4.5	*Temp_s_var_2050_45*	*Stand. dev.*	8.66	5.26	0.33	22.77	Grid-cell	Y
	Seasonal variability 2050 rcp 8.5	*Temp_s_var_2050_85*	*Stand. dev.*	8.66	5.24	0.33	22.95	Grid-cell	Y
	Seasonal variability 2100 rcp 4.5	*Temp_s_var_2100_45*	*Stand. dev.*	8.57	5.16	0.33	22.64	Grid-cell	Y
	Seasonal variability 2100 rcp 8.5	*Temp_s_var_2100_85*	*Stand. dev.*	8.45	4.85	0.36	21.75	Grid-cell	Y
**Projected Scenarios - Population**									
Population	Population density 2050	*Pop_density_2050*	*People sqkm^−1^*	50	269	0	22,622	Grid-cell	Y
	Population density 2100	*Pop_density_2100*	*People sqkm^−1^*	55	315	0	29,243	Grid-cell	Y

**Table A2:** Main data used for the analysis.

Variable	Spatial coverage / resolution	Temporal coverage / resolution	Database	Reference	URL
Hydro-political interaction	Global /Basin-Country_Unit (BCU)	1997-2007	International River Cooperation and Conflict IRCC	Kalbhenn and Bernauer, 2012	http://www.ib.ethz.ch/data.html
World river basins	Global / Spatial polygon	–	International River Dataset	Beck et al., 2014	http://ir-s01.ethz.ch/
Climate forcing	Global / 0.25 degrees	1979 – 2015 / 3 hours	Multi-Source Weighted-Ensemble Precipitation (MSWEP)	Beck et al., 2017	http://www.gloh2o.org/
Climate forcing	Global / 0.25 degrees	1979 – 2015 / 3 hours	WATCH Forcing Data methodology applied to ERA-Interim (WFDEI)	Weedon et al., 2014	ftp://rfdata:forceDATA@ftp.iiasa.ac.at
Gross Domestic Product (GDP)	Global / Country	1950-2011 / year	Expanded GDP data – Version 6.0	Gleditsch, 2002	http://privatewww.essex.ac.uk/∼ksg/exptradegdp.html
Governance indicator	Global / Country	1996-2015 / year	Worldwide Governance Indicators (WGI)	Kaufmann et al., 2010	http://info.worldbank.org/governance/wgi/#home
Agriculture (% GDP) and Rural population (% of the total)	Global / Country	1960-2016 / year	World Development Indicator (WDI) database	World Bank 2017 (accessed in February 2017)	http://data.worldbank.org/data-catalog/world-development-indicators
Population	Global / 1 km	Multitemporal (1975, 1990, 2000, 2015)	GHS population grid, derived from the Gridded Population of the World (GPW, v4)	Freire and Pesaresi, 2015 – CIESIN, 2015	http://data.europa.eu/89h/jrc-ghsl-ghs_pop_gpw4_globe_r2015a http://ghsl.jrc.ec.europa.eu/ghs_pop.php
Composite Index of National Capability (CINC)	Global / Country	1816-2007 / year	National Material Capability (NMC) version 5.0 – Correlates of War (CoW)	Singer et al., 1972	http://www.correlatesofwar.org/data-sets/national-material-capabilities
International Freshwater Treaty Database – IFTD	Global / BCU	1820 - 2007	International Freshwater Treaty Database – IFTD - Transboundary Freshwater Dispute Database TFDD	De Stefano et al., 2012	http://www.transboundarywaters.orst.edu/database/interfreshtreatdata.html
Climate future scenarios	Global / 0.25 degrees	1950 – 2100 / day	NASA Earth Exchange Global Daily Downscaled Projections (NEX-GDDP) dataset	Thrasher et al., 2012	https://nex.nasa.gov/nex/projects/1356/ https://dataserver.nccs.nasa.gov/thredds/catalog/bypass/NEX-GDDP/catalog.html
Population trends	Global / Country	1950 – 2100 / 5 years	United Nations - Department of Economic and Social Affairs, Population Division	UN/DESA, 2017	https://esa.un.org/unpd/wpp/

**Fig. A1:**
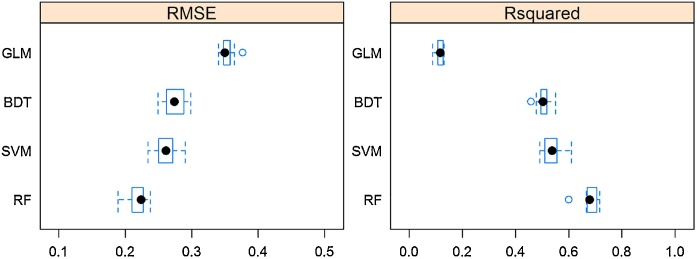
10-k cross validation: comparison between Generalized Linear Model (GLM), Boosted Decision Trees (BDT), and Support Vector Machine (SVM). Lower values of RMSE and higher values of R-squared are preferred.

**Fig. A2:**
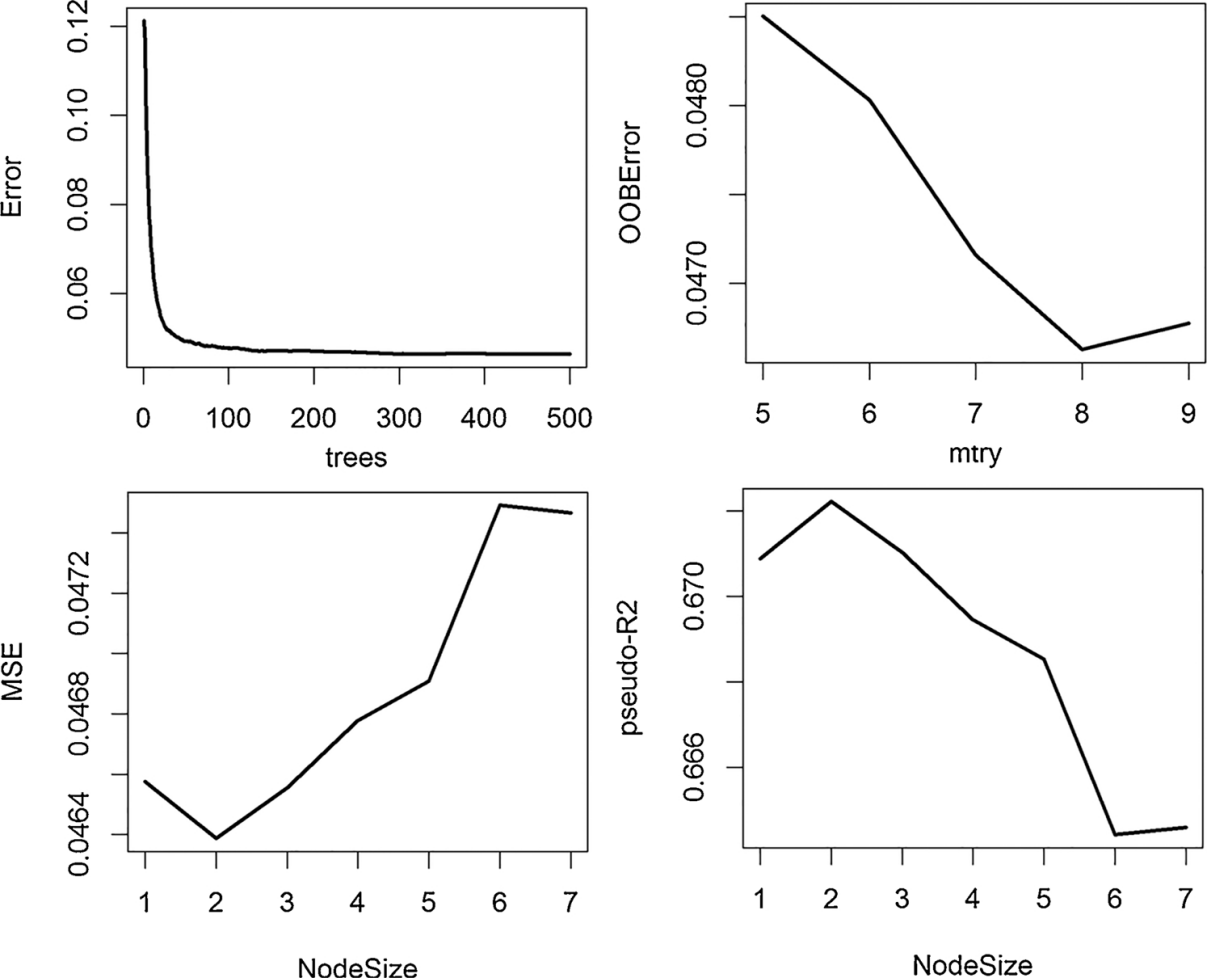
Parameter calibration – top: left: *ntree* vs *MSE*; right: *mtry* vs Out of Bag Error *OOB-MSE*; bottom: NodeSize vs MSE (left), and NodeSize vs Pseudo R2 (right).

**Fig. A6:**
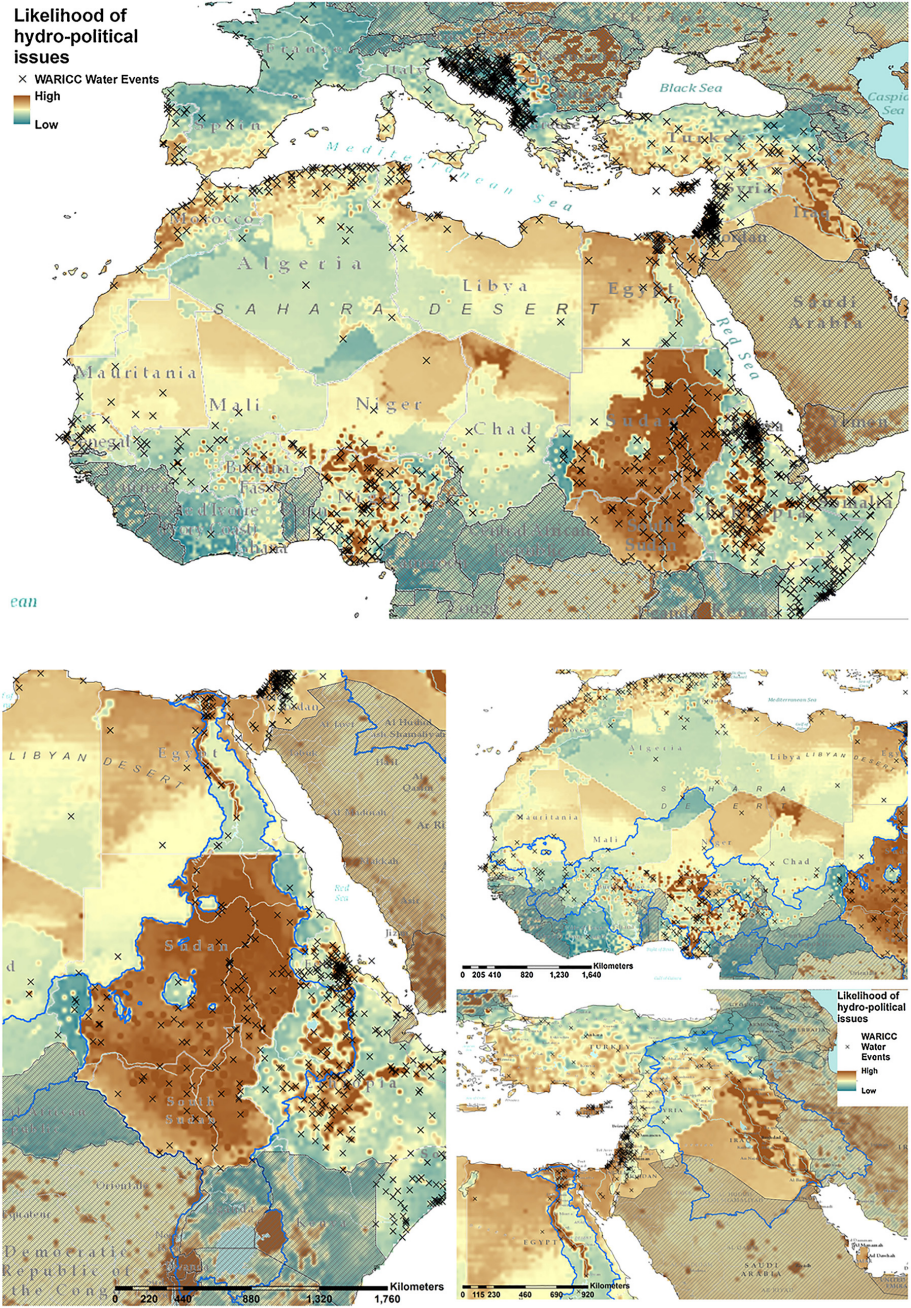
Comparison between water events reported in the WARICC database between 1997 and 2009 (Bernauer et al., 2012a) and the likelihood of hydro-political interactions presented in this study (shaded areas are not considered in the WARICC database).

**Table A3:** Normalized likelihood of hydro-political interaction, future scenarios, and main determining factors per river basin.

Basin_ID	National Power (CINC)	Governance	Economic Wealth	Economic dependence on agriculture	International treaties in the basin	Population Density	Population in Rural Areas	Water availability	Precipitation stress	Yearly precipitation	Climatic zone (Temperature)	Likelihood hydro-polit issues_norm	likelihood_bline	% change_2050_RCP4.5_vs_bline	% change_2050_RCP8.5_vs_bline	% change_2100_RCP4.5_vs_bline	% change_2100_RCP8.5_vs_bline	Name of the Basin	no.
131	0.914	0.369	0.370	0.737	0.625	0.942	0.794	0.234	0.514	0.672	0.726	1.000	0.535	7.177	7.143	6.657	7.740	Ganges/Brahmaputra	1
135	1.000	0.330	0.495	0.602	0.623	0.865	0.649	0.195	0.629	0.702	0.764	0.999	0.535	9.735	10.721	7.268	9.612	Pearl	2
57	0.984	0.858	0.942	0.061	0.999	0.506	0.160	0.440	0.527	0.504	0.552	0.799	0.436	37.895	38.335	39.870	42.001	Mississippi	3
130	0.516	0.129	0.262	0.882	0.420	0.675	0.806	0.394	0.518	0.442	0.933	0.761	0.418	43.399	43.142	45.220	42.469	Nile	4
248	0.776	0.482	0.588	0.384	0.693	0.674	0.125	0.626	0.533	0.638	0.715	0.756	0.415	−8.030	−8.225	−37.233	−30.656	Chuy	5
141	0.847	0.338	0.294	0.736	0.600	0.963	0.821	0.297	0.490	0.886	0.911	0.679	0.377	30.565	30.362	31.508	31.298	Feni	6
114	0.837	0.239	0.341	0.765	0.472	0.832	0.756	0.242	0.621	0.469	0.623	0.675	0.375	12.325	12.571	15.398	19.152	Indus	7
82	0.985	0.854	0.941	0.062	1.000	0.416	0.160	0.425	0.529	0.252	0.570	0.670	0.373	19.048	20.118	21.261	25.488	Colorado	8
84	0.999	0.327	0.494	0.610	0.624	0.400	0.647	0.208	0.527	0.034	0.478	0.658	0.367	12.355	13.503	14.840	18.797	Tarim	9
104	0.647	0.191	0.593	0.491	0.412	0.619	0.307	0.330	0.634	0.283	0.770	0.592	0.335	23.191	23.550	26.545	32.532	Shatt al-Arab - tigris/Euphrates	10
110	0.513	0.101	0.558	0.715	0.363	0.495	0.585	0.734	0.422	0.181	0.682	0.577	0.327	16.011	16.935	15.378	17.544	Hari	11
132	0.735	0.050	0.393	0.938	0.280	0.661	0.802	0.529	0.475	0.745	0.827	0.573	0.326	26.150	26.687	26.387	28.578	Irrawaddy	12
44	0.630	0.623	0.697	0.318	0.667	0.779	0.385	0.170	0.286	0.429	0.492	0.566	0.322	13.420	14.818	3.903	21.293	Vistula	13
79	0.847	0.272	0.669	0.400	0.688	0.704	0.254	0.629	0.407	0.541	0.465	0.565	0.321	14.538	15.129	16.204	24.305	Terek	14
245	0.255	0.337	0.347	0.664	0.535	0.583	0.861	0.420	0.246	0.483	0.832	0.564	0.321	49.606	48.309	61.302	55.689	Umbeluzi	15
51	0.967	0.871	0.936	0.069	0.976	0.391	0.160	0.579	0.367	0.509	0.447	0.543	0.311	22.426	23.327	26.039	33.005	Columbia	16
123	0.345	0.332	0.596	0.385	0.262	0.932	0.074	0.011	0.523	0.518	0.702	0.538	0.308	24.587	25.240	21.536	33.438	Wadi Al Izziyah	17
242	0.545	0.578	0.539	0.352	0.666	0.438	0.518	0.571	0.212	0.253	0.750	0.522	0.300	33.894	33.924	35.468	39.138	Orange	18
233	0.305	0.506	0.552	0.416	0.460	0.180	0.600	0.665	0.036	0.380	0.834	0.521	0.300	22.035	22.276	23.819	24.930	Okavango	19
143	0.929	0.329	0.432	0.678	0.535	0.816	0.740	0.188	0.654	0.847	0.835	0.515	0.297	39.760	39.426	33.798	34.072	Ka Long	20
107	0.647	0.177	0.607	0.575	0.476	0.562	0.403	0.665	0.390	0.348	0.655	0.506	0.293	28.520	29.178	29.114	30.587	Atrek	21
120	0.519	0.000	0.273	0.819	0.236	0.497	0.828	0.404	0.538	0.118	0.713	0.505	0.292	30.626	31.593	32.292	40.272	Sistan Basin	22
244	0.560	0.498	0.473	0.565	0.682	0.599	0.568	0.393	0.267	0.498	0.796	0.503	0.291	34.967	34.728	39.035	40.384	Komati	23
59	0.568	0.584	0.719	0.471	0.588	0.720	0.421	0.621	0.434	0.504	0.524	0.499	0.289	24.236	26.075	19.280	34.751	Danube	24
246	0.546	0.469	0.502	0.435	0.675	0.622	0.610	0.279	0.408	0.493	0.766	0.492	0.286	44.019	43.416	46.850	47.083	Maputo	25
122	0.836	0.301	0.435	0.793	0.464	0.671	0.781	0.741	0.496	0.710	0.818	0.492	0.285	25.191	25.722	23.442	24.132	Mekong	26
118	0.547	0.413	0.402	0.640	0.161	0.751	0.475	0.043	0.553	0.250	0.636	0.486	0.283	40.140	39.405	38.553	47.336	Bon Naima	27
35	0.939	0.306	0.605	0.521	0.658	0.551	0.420	0.546	0.660	0.427	0.273	0.486	0.283	22.040	22.141	22.208	30.770	Amur	28
237	0.339	0.409	0.426	0.520	0.472	0.345	0.717	0.736	0.000	0.368	0.830	0.474	0.277	32.909	33.264	40.085	39.081	Etosha pan	29
112	0.408	0.020	0.418	0.802	0.241	0.460	0.772	0.646	0.598	0.309	0.647	0.463	0.271	27.397	28.805	26.423	40.916	Morghab	30
171	0.550	0.206	0.029	0.968	0.411	0.700	0.973	0.248	0.639	0.427	0.866	0.456	0.268	48.466	48.526	53.271	52.514	Lake Abbe	31
64	0.486	0.209	0.532	0.683	0.587	0.519	0.608	0.551	0.442	0.265	0.516	0.455	0.268	33.255	34.251	35.684	41.908	Aral Sea	32
106	0.750	0.453	0.734	0.720	0.119	0.999	0.239	0.173	0.414	0.683	0.533	0.453	0.266	39.803	40.756	43.653	51.858	Han	33
121	0.527	0.384	0.547	0.687	0.379	0.800	0.270	0.062	0.606	0.043	0.744	0.452	0.266	34.974	35.191	36.876	38.270	Jordan/Dead Sea	34
139	0.497	0.268	0.187	0.865	0.544	0.598	0.757	0.295	0.482	0.425	0.975	0.447	0.263	64.156	62.392	76.026	66.048	Niger	35
164	0.323	0.365	0.300	0.590	0.413	0.900	0.488	0.307	0.429	0.727	0.827	0.439	0.260	42.833	39.332	38.346	38.480	Paz	36
241	0.525	0.485	0.522	0.537	0.631	0.598	0.557	0.481	0.318	0.380	0.816	0.438	0.259	37.244	36.743	39.585	41.529	Limpopo	37
239	0.367	0.148	0.454	0.717	0.420	0.572	0.759	0.569	0.162	0.441	0.807	0.438	0.259	32.701	33.190	37.219	33.807	Save	38
158	0.693	0.450	0.664	0.279	0.876	0.906	0.207	0.297	0.429	0.798	0.854	0.435	0.258	36.891	36.078	35.299	40.257	Coatan	39
133	0.691	0.209	0.615	0.476	0.521	0.476	0.323	0.268	0.546	0.087	0.895	0.433	0.256	21.881	20.925	19.092	19.746	Bahu Kalat	40
228	0.504	0.027	0.184	0.758	0.324	0.630	0.692	0.798	0.368	0.648	0.896	0.432	0.256	76.422	72.838	89.122	86.112	Chiloango	41
188	0.487	0.055	0.087	0.830	0.329	0.486	0.690	0.787	0.464	0.688	0.882	0.432	0.256	70.974	71.093	78.190	83.396	Congo	42
231	0.374	0.281	0.312	0.688	0.428	0.520	0.752	0.617	0.123	0.550	0.839	0.431	0.256	38.997	38.543	48.453	47.166	Zambezi	43
95	0.934	0.193	0.409	0.851	0.509	0.717	0.532	0.205	0.769	0.522	0.391	0.431	0.256	49.424	49.336	54.487	63.095	Yalu	44
119	0.345	0.332	0.596	0.385	0.262	0.907	0.074	0.154	0.292	0.474	0.741	0.431	0.255	41.130	42.978	42.289	50.967	Nahr al-Kabir al-Janoubi	45
203	0.594	0.525	0.600	0.529	0.317	0.794	0.520	0.484	0.244	0.882	0.949	0.430	0.255	56.644	56.191	58.877	63.133	Golok	46
240	0.367	0.362	0.123	0.809	0.527	0.485	0.795	0.558	0.231	0.576	0.842	0.429	0.255	44.165	45.605	57.977	57.144	Buzi	47
108	0.632	0.293	0.525	0.711	0.495	0.807	0.420	0.175	0.365	0.363	0.672	0.427	0.254	43.776	45.252	50.121	62.190	Orontes	48
155	0.484	0.149	0.137	0.839	0.290	0.643	0.899	0.257	0.343	0.330	0.918	0.425	0.253	44.890	45.321	55.501	54.110	Mareb	49
234	0.444	0.169	0.394	0.497	0.366	0.465	0.722	0.699	0.052	0.487	0.768	0.421	0.251	29.415	28.827	38.116	32.273	Cunene	50
111	0.923	0.669	0.855	0.181	0.951	0.524	0.182	0.379	0.601	0.295	0.716	0.419	0.250	35.540	36.125	36.227	39.494	Grande	51
124	0.894	0.613	0.820	0.210	0.934	0.545	0.188	0.362	0.426	0.228	0.628	0.417	0.249	44.239	43.658	43.027	42.764	Tijuana	52
185	0.509	0.227	0.129	0.915	0.393	0.667	0.941	0.245	0.387	0.563	0.867	0.416	0.248	50.010	50.492	58.676	59.113	Lake Turkana	53
138	0.798	0.161	0.370	0.892	0.475	0.491	0.810	0.478	0.449	0.839	0.808	0.415	0.248	27.036	27.538	26.590	32.981	Kaladan	54
56	0.394	0.457	0.413	0.762	0.320	0.147	0.382	0.594	0.776	0.085	0.212	0.413	0.247	12.058	12.288	13.367	23.324	Khyargas Nuur	55
58	0.881	0.910	0.915	0.094	0.882	0.551	0.158	0.744	0.577	0.568	0.403	0.412	0.246	30.829	32.246	37.467	55.786	Saint Lawrence	56
86	0.843	0.274	0.665	0.416	0.680	0.556	0.263	0.619	0.258	0.570	0.365	0.410	0.245	6.540	8.538	11.796	30.443	Sulak	57
116	0.668	0.161	0.573	0.594	0.480	0.364	0.436	0.321	0.590	0.208	0.723	0.410	0.245	36.506	41.215	37.430	49.433	Kowl-e Namakzar	58
125	0.929	0.203	0.463	0.804	0.499	0.552	0.720	0.451	0.396	0.649	0.600	0.405	0.243	11.027	11.374	9.907	12.980	Salween	59
19	0.853	0.268	0.676	0.373	0.700	0.605	0.241	0.629	0.500	0.450	0.399	0.402	0.242	14.911	17.514	16.818	38.846	Volga	60
12	0.832	0.274	0.664	0.393	0.692	0.373	0.287	0.616	0.468	0.401	0.301	0.400	0.241	20.203	20.882	22.444	34.406	Ob	61
2	0.841	0.287	0.661	0.445	0.680	0.206	0.255	0.626	0.426	0.415	0.167	0.399	0.240	15.028	15.150	16.938	21.381	Yenisei	62
117	0.572	0.256	0.454	0.546	0.207	0.823	0.389	0.000	0.562	0.252	0.664	0.398	0.239	60.379	60.820	61.724	69.363	Tafna	63
173	0.586	0.295	0.314	0.812	0.359	0.909	0.874	0.479	0.402	0.733	0.973	0.397	0.239	52.008	53.182	54.810	54.593	Vam Co	64
115	0.657	0.323	0.563	0.688	0.498	0.695	0.406	0.154	0.237	0.537	0.686	0.393	0.237	60.662	60.731	60.745	73.029	Nahr El Kebir	65
223	0.338	0.294	0.288	0.898	0.161	0.359	0.997	0.792	0.181	0.908	0.913	0.391	0.236	66.842	69.560	72.281	73.869	Sepik	66
140	0.802	0.300	0.265	0.736	0.552	0.966	0.828	0.344	0.527	0.891	0.904	0.381	0.231	64.706	65.589	66.616	64.365	Karnaphuli	67
136	0.938	0.328	0.438	0.674	0.545	0.791	0.731	0.417	0.534	0.684	0.796	0.380	0.231	44.530	45.087	45.591	53.619	Red	68
92	0.578	0.507	0.654	0.529	0.487	0.654	0.286	0.680	0.357	0.450	0.583	0.380	0.230	50.216	53.315	44.838	59.882	Maritsa	69
215	0.587	0.333	0.553	0.513	0.090	0.658	0.242	0.721	0.219	0.871	0.765	0.377	0.229	40.394	41.311	39.038	47.912	Patia	70
152	0.457	0.134	0.160	0.790	0.243	0.561	0.877	0.276	0.333	0.173	0.951	0.377	0.229	43.335	44.161	54.466	57.474	Baraka	71
229	0.319	0.294	0.286	0.900	0.161	0.187	1.000	0.792	0.248	0.938	0.910	0.374	0.228	69.094	72.969	78.131	77.008	Fly	72
243	0.579	0.418	0.654	0.466	0.627	0.000	0.029	0.720	0.602	0.000	0.393	0.372	0.227	38.994	37.835	58.107	73.794	Laguna de Tara	73
148	0.261	0.352	0.197	0.839	0.554	0.443	0.655	0.403	0.281	0.351	1.000	0.372	0.227	36.799	36.410	45.407	41.005	Senegal	74
113	0.465	0.387	0.521	0.536	0.217	0.736	0.359	0.006	0.202	0.375	0.699	0.371	0.226	63.119	63.409	62.729	66.377	Medjerda	75
49	0.842	0.273	0.662	0.409	0.688	0.591	0.250	0.609	0.423	0.395	0.490	0.370	0.226	46.478	51.039	50.766	67.073	Don	76
161	0.329	0.396	0.194	0.889	0.405	0.623	0.741	0.273	0.439	0.546	0.977	0.369	0.225	81.633	79.409	96.946	93.278	Volta	77
159	0.345	0.308	0.431	0.608	0.490	0.924	0.580	0.317	0.375	0.812	0.779	0.367	0.224	52.838	52.184	51.782	49.953	Suchiate	78
144	0.559	0.266	0.316	0.843	0.400	0.687	0.836	0.831	0.651	0.715	0.851	0.366	0.224	44.895	46.094	45.062	49.497	Ma	79
80	0.963	0.241	0.444	0.786	0.556	0.683	0.571	0.311	0.800	0.449	0.356	0.354	0.218	51.331	52.762	52.802	69.604	Tumen	80
145	0.589	0.287	0.323	0.813	0.397	0.742	0.835	0.805	0.711	0.725	0.862	0.353	0.217	59.430	60.639	60.561	61.692	Ca	81
154	0.131	0.457	0.512	0.647	0.116	0.393	0.587	0.715	0.627	0.756	0.899	0.350	0.216	53.080	54.425	55.682	65.549	Belize	82
129	0.763	0.480	0.703	0.268	0.888	0.275	0.203	0.304	0.601	0.367	0.756	0.350	0.216	51.042	50.632	50.584	49.151	Yaqui	83
24	0.174	0.743	0.710	0.302	0.333	0.436	0.300	0.516	0.432	0.471	0.445	0.350	0.216	26.681	32.136	27.052	45.597	Parnu	84
182	0.486	0.119	0.132	0.948	0.340	0.528	0.891	0.265	0.577	0.361	0.910	0.348	0.215	51.620	52.139	60.391	61.988	Jubba	85
162	0.299	0.380	0.220	0.596	0.327	0.788	0.464	0.344	0.414	0.735	0.862	0.344	0.213	65.255	65.715	64.319	67.353	Lempa	86
55	0.692	0.409	0.515	0.702	0.482	0.126	0.347	0.603	0.640	0.204	0.215	0.344	0.213	27.674	27.868	28.422	40.177	Uvs Nuur	87
235	0.694	0.433	0.591	0.499	0.642	0.551	0.147	0.750	0.522	0.626	0.816	0.340	0.211	61.130	61.532	61.541	65.097	La Plata	88
163	0.288	0.364	0.211	0.718	0.546	0.540	0.654	0.430	0.390	0.510	0.987	0.340	0.211	45.265	45.516	53.507	50.611	Gambia	89
249	0.512	0.801	0.661	0.341	0.262	0.573	0.065	0.580	0.533	0.731	0.564	0.339	0.210	62.603	65.476	66.910	77.887	Itata	90
48	0.637	0.678	0.725	0.253	0.689	0.766	0.379	0.109	0.300	0.424	0.511	0.336	0.209	26.985	29.252	25.959	49.819	Oder	91
66	0.777	0.294	0.585	0.522	0.630	0.840	0.289	0.536	0.420	0.404	0.529	0.336	0.209	66.922	70.718	68.196	79.323	Mius	92
232	0.398	0.372	0.068	0.838	0.521	0.448	0.825	0.510	0.354	0.595	0.868	0.335	0.208	70.740	70.465	74.673	74.437	Ruvuma	93
146	0.354	0.379	0.560	0.448	0.363	0.889	0.323	0.119	0.168	0.740	0.868	0.335	0.208	39.982	38.825	37.597	46.008	Dajabon	94
77	0.337	0.544	0.712	0.385	0.262	0.601	0.459	0.621	0.397	0.531	0.630	0.332	0.207	40.074	38.096	30.458	44.491	Krka	95
214	0.157	0.329	0.613	0.383	0.165	0.201	0.163	0.823	0.508	0.744	0.901	0.332	0.207	61.174	63.429	67.555	74.938	Ogooue	96
254	0.512	0.801	0.661	0.341	0.262	0.298	0.065	0.586	0.366	0.702	0.428	0.330	0.206	16.922	14.697	26.705	71.237	Aysen	97
166	0.282	0.359	0.271	0.616	0.278	0.652	0.497	0.431	0.155	0.716	0.848	0.329	0.205	66.726	69.933	67.155	70.093	Goascoran	98
178	0.574	0.247	0.584	0.417	0.213	0.366	0.119	0.743	0.276	0.824	0.917	0.329	0.205	54.367	55.484	56.799	61.101	Orinoco	99
109	0.691	0.209	0.615	0.476	0.521	0.599	0.323	0.279	0.821	0.519	0.609	0.325	0.203	148.591	149.671	139.922	145.642	Astara	100
127	0.555	0.365	0.420	0.614	0.176	0.442	0.448	0.035	0.639	0.112	0.759	0.324	0.203	45.242	44.446	44.398	40.608	Daoura	101
99	0.844	0.265	0.667	0.394	0.684	0.531	0.260	0.601	0.261	0.539	0.350	0.318	0.200	21.566	23.694	28.921	47.519	Samur	102
76	0.965	0.305	0.588	0.526	0.659	0.602	0.483	0.538	0.757	0.439	0.379	0.318	0.200	55.322	57.039	58.810	85.333	Razdolnaya	103
169	0.217	0.244	0.150	0.897	0.406	0.603	0.667	0.504	0.392	0.618	0.969	0.316	0.199	67.153	69.001	71.816	66.659	Geba	104
184	0.579	0.281	0.572	0.459	0.171	0.650	0.167	0.737	0.123	0.816	0.885	0.315	0.198	62.539	62.080	61.424	66.345	Catatumbo	105
206	0.726	0.436	0.557	0.458	0.637	0.280	0.183	0.653	0.407	0.818	0.905	0.314	0.198	69.531	71.949	74.245	79.205	Amazon	106
205	0.655	0.557	0.668	0.461	0.000	0.114	0.339	0.917	0.339	0.834	0.922	0.314	0.198	83.329	81.464	82.615	97.463	Maroni	107
238	0.425	0.481	0.505	0.578	0.413	0.131	0.279	0.682	0.418	0.225	0.464	0.314	0.198	34.925	36.805	38.625	49.510	Salar de Coipasa	108
194	0.395	0.340	0.412	0.750	0.091	0.103	0.623	0.952	0.344	0.799	0.918	0.314	0.198	90.354	93.346	95.117	94.494	Essequibo	109
30	0.794	0.929	0.905	0.105	0.823	0.304	0.157	0.785	0.420	0.390	0.344	0.313	0.198	43.446	42.903	47.988	71.267	Nelson	110
105	0.660	0.775	0.832	0.257	0.734	0.517	0.236	0.212	0.332	0.347	0.681	0.309	0.196	55.291	56.500	55.106	57.243	Guadiana	111
137	0.455	0.146	0.163	0.942	0.463	0.588	0.796	0.622	0.618	0.451	0.959	0.308	0.195	77.261	76.721	85.745	78.406	Lake Chad	112
227	0.477	0.337	0.512	0.540	0.552	0.618	0.303	0.591	0.154	0.517	0.763	0.300	0.191	78.424	79.710	79.433	78.167	Chira	113
251	0.579	0.418	0.654	0.466	0.627	0.000	0.029	0.580	0.511	0.927	0.395	0.298	0.190	29.415	27.369	29.453	67.673	Reremo	114
10	0.936	0.889	0.927	0.081	0.938	0.021	0.159	0.674	0.638	0.394	0.161	0.288	0.186	12.881	12.881	12.410	13.457	Yukon	115
128	0.554	0.370	0.418	0.617	0.175	0.362	0.451	0.035	0.659	0.102	0.782	0.286	0.184	45.867	44.195	42.345	36.756	Draa	116
40	0.813	0.401	0.687	0.341	0.693	0.613	0.292	0.482	0.383	0.466	0.477	0.284	0.183	35.546	38.240	38.994	54.089	Pregolya	117
247	0.668	0.599	0.612	0.467	0.646	0.385	0.045	0.879	0.471	0.668	0.724	0.284	0.183	86.172	87.663	88.132	90.378	Lagoa Mirim	118
172	0.633	0.328	0.336	0.742	0.389	0.964	0.835	0.390	0.404	0.823	0.916	0.278	0.180	89.899	91.832	93.084	95.622	Saigon	119
68	0.821	0.281	0.636	0.457	0.668	0.391	0.265	0.487	0.388	0.402	0.538	0.274	0.179	103.114	105.280	100.864	114.788	Elancik	120
175	0.613	0.198	0.474	0.849	0.307	0.676	0.768	0.539	0.417	0.917	0.935	0.271	0.177	99.318	98.860	100.004	111.787	Kraburi	121
134	0.696	0.202	0.414	0.745	0.360	0.451	0.659	0.174	0.586	0.049	0.896	0.270	0.177	39.833	39.831	43.966	50.476	Dasht	122
179	0.424	0.378	0.192	0.830	0.479	0.814	0.673	0.385	0.427	0.619	0.961	0.269	0.176	90.197	89.592	98.651	94.495	Oueme	123
33	0.679	0.940	0.898	0.111	0.780	0.307	0.157	0.804	0.336	0.580	0.363	0.266	0.174	15.388	17.901	22.999	50.747	Fraser	124
94	0.435	0.521	0.629	0.532	0.473	0.280	0.279	0.746	0.503	0.431	0.591	0.265	0.174	86.589	86.945	94.064	101.435	Veleka	125
258	0.553	0.591	0.657	0.416	0.520	0.290	0.045	0.635	0.459	0.258	0.454	0.260	0.171	46.802	48.582	50.719	59.267	Gallegos	126
15	0.825	0.428	0.738	0.349	0.699	0.491	0.215	0.612	0.515	0.471	0.392	0.259	0.171	1.093	5.871	6.675	39.432	Neva	127
43	0.787	0.467	0.693	0.324	0.690	0.582	0.318	0.102	0.304	0.507	0.476	0.256	0.170	37.708	38.605	42.487	63.132	Prokhladnaya	128
31	0.261	0.642	0.684	0.349	0.413	0.612	0.319	0.467	0.467	0.463	0.463	0.254	0.169	36.106	38.221	41.699	57.345	Lielupe	129
253	0.546	0.632	0.658	0.403	0.486	0.066	0.049	0.612	0.281	0.801	0.452	0.254	0.169	33.442	33.457	38.158	90.112	Palena	130
126	0.570	0.271	0.449	0.556	0.203	0.269	0.397	0.010	0.573	0.006	0.788	0.254	0.169	43.824	43.493	42.999	48.813	Guir	131
189	0.185	0.510	0.633	0.391	0.217	0.541	0.371	0.586	0.255	0.981	0.683	0.250	0.167	55.589	56.549	56.286	64.103	Chiriqui Viejo	132
93	0.586	0.299	0.548	0.621	0.328	0.678	0.418	0.354	0.424	0.465	0.519	0.249	0.166	94.790	98.658	102.868	123.806	Kura	133
252	0.561	0.539	0.657	0.432	0.557	0.090	0.041	0.650	0.384	0.814	0.460	0.249	0.166	47.315	46.236	57.048	93.187	Yelcho	134
36	0.736	0.278	0.592	0.543	0.596	0.646	0.285	0.445	0.417	0.438	0.487	0.248	0.166	77.638	82.214	80.318	115.561	Dnieper	135
261	0.550	0.609	0.658	0.410	0.506	0.308	0.047	0.634	0.053	0.249	0.444	0.243	0.163	45.337	52.485	58.706	59.204	Chorrillo Gama	136
256	0.549	0.616	0.658	0.408	0.500	0.023	0.048	0.638	0.499	0.569	0.422	0.243	0.163	38.084	37.882	41.435	69.739	Pascua	137
7	0.894	0.906	0.917	0.091	0.894	0.000	0.158	0.731	0.595	0.255	0.000	0.240	0.162	41.525	42.947	43.800	37.571	Firth	138
62	0.969	0.869	0.937	0.068	0.979	0.266	0.160	0.566	0.280	0.833	0.391	0.240	0.162	22.857	25.265	29.616	67.280	Skagit	139
69	0.736	0.686	0.870	0.190	0.724	0.875	0.311	0.129	0.610	0.585	0.533	0.238	0.161	41.556	41.986	45.016	64.626	Po	140
41	0.773	0.283	0.642	0.422	0.678	0.433	0.363	0.594	0.500	0.286	0.428	0.238	0.161	75.811	80.973	85.096	115.802	Ural	141
236	0.444	0.374	0.456	0.580	0.514	0.442	0.304	0.691	0.300	0.404	0.473	0.236	0.160	59.399	61.091	63.889	74.703	Lake Poopo	142
63	0.975	0.359	0.480	0.644	0.582	0.258	0.586	0.402	0.408	0.172	0.351	0.235	0.159	70.909	72.570	77.699	103.299	Ulungur Lake	143
250	0.556	0.571	0.657	0.422	0.535	0.371	0.044	0.654	0.527	0.865	0.441	0.235	0.159	66.619	64.372	72.727	111.168	Puelo	144
90	0.472	0.536	0.700	0.500	0.490	0.700	0.298	0.629	0.293	0.449	0.547	0.229	0.157	94.276	100.482	104.004	136.217	Struma	145
218	0.097	0.236	0.662	0.251	0.090	0.191	0.403	0.763	0.641	0.847	0.898	0.228	0.156	120.453	117.838	118.462	132.059	Muni	146
61	0.668	0.324	0.488	0.625	0.536	0.738	0.391	0.319	0.407	0.429	0.515	0.226	0.155	90.221	101.880	97.406	133.495	Dniester	147
262	0.556	0.571	0.657	0.422	0.535	0.044	0.044	0.624	0.119	0.253	0.430	0.226	0.155	31.452	33.336	40.908	54.445	Carmen Sylva	148
222	0.167	0.304	0.590	0.373	0.146	0.153	0.191	0.855	0.347	0.730	0.905	0.220	0.152	101.254	101.497	103.809	117.353	Nyanga	149
102	0.657	0.776	0.830	0.256	0.735	0.723	0.243	0.219	0.396	0.439	0.640	0.220	0.152	90.515	91.062	90.375	96.143	Tagus	150
81	0.292	0.373	0.557	0.555	0.033	0.601	0.648	0.515	0.286	0.664	0.532	0.217	0.151	71.715	74.709	72.156	108.972	Neretva	151
201	0.595	0.174	0.199	0.848	0.498	0.777	0.655	0.322	0.359	0.817	0.935	0.213	0.149	122.078	121.610	135.796	134.107	Cross	152
42	0.650	0.906	0.907	0.094	0.675	0.552	0.309	0.256	0.673	0.595	0.505	0.213	0.148	18.451	18.818	19.793	23.536	Erne	153
255	0.532	0.705	0.659	0.377	0.413	0.060	0.056	0.612	0.395	0.523	0.429	0.211	0.147	28.357	24.239	33.961	57.870	Baker	154
221	0.706	0.286	0.406	0.648	0.161	0.261	0.600	0.423	0.336	0.882	0.908	0.210	0.147	122.709	126.033	128.722	138.947	Mamberamo	155
151	0.639	0.397	0.603	0.446	0.790	0.645	0.345	0.309	0.460	0.765	0.881	0.209	0.147	101.391	102.178	105.692	115.989	Grijalva	156
165	0.271	0.318	0.364	0.642	0.262	0.850	0.564	0.431	0.315	0.655	0.863	0.208	0.146	96.493	98.875	97.382	105.066	Choluteca	157
97	0.496	0.585	0.730	0.461	0.543	0.504	0.257	0.659	0.231	0.488	0.515	0.208	0.146	99.893	117.166	123.834	150.927	Nestos	158
8	0.841	0.344	0.708	0.362	0.700	0.440	0.228	0.622	0.386	0.458	0.285	0.207	0.146	−3.351	−3.359	−3.072	3.904	Tuloma	159
220	0.435	0.302	0.177	0.846	0.431	0.526	0.889	0.293	0.448	0.506	0.802	0.206	0.145	101.007	94.896	111.627	109.755	Lake Natron	160
170	0.231	0.155	0.150	0.842	0.514	0.499	0.735	0.460	0.433	0.724	0.946	0.204	0.144	112.089	111.106	117.249	108.119	Corubal	161
216	0.517	0.300	0.524	0.566	0.403	0.606	0.331	0.625	0.285	0.855	0.725	0.204	0.144	68.638	69.153	70.800	72.413	Mira	162
20	0.925	0.894	0.924	0.084	0.925	0.053	0.159	0.748	0.388	0.673	0.275	0.202	0.143	10.037	10.291	13.495	34.315	Chilkat	163
225	0.454	0.308	0.507	0.569	0.531	0.593	0.347	0.583	0.229	0.613	0.819	0.195	0.140	130.927	130.265	126.865	124.303	Tumbes	164
89	0.676	0.772	0.841	0.260	0.726	0.599	0.190	0.176	0.471	0.477	0.591	0.191	0.138	79.343	81.642	84.993	106.493	Ebro	165
259	0.512	0.801	0.661	0.341	0.262	0.478	0.065	0.580	0.057	0.254	0.450	0.191	0.138	32.763	30.245	35.212	38.374	Cullen	166
263	0.553	0.588	0.657	0.417	0.523	0.197	0.045	0.623	0.294	0.302	0.428	0.188	0.136	43.748	46.420	50.323	63.073	Grande	167
230	0.656	0.289	0.372	0.760	0.161	0.347	0.738	0.651	0.270	0.800	0.948	0.188	0.136	127.423	143.191	158.373	154.442	Maro	168
71	0.623	0.334	0.453	0.663	0.481	0.512	0.457	0.278	0.444	0.344	0.560	0.187	0.136	142.102	149.462	125.675	181.356	Sarata	169
212	0.000	0.428	0.436	0.665	0.058	0.008	0.506	1.000	0.278	0.823	0.926	0.185	0.135	174.396	182.228	195.985	195.665	Courantyne	170
183	0.185	0.510	0.633	0.391	0.217	0.573	0.371	0.586	0.135	0.958	0.733	0.185	0.135	91.256	90.034	86.362	121.519	Changuinola	171
70	0.635	0.331	0.461	0.655	0.495	0.639	0.441	0.331	0.354	0.364	0.555	0.184	0.135	143.751	154.680	144.589	190.962	Kogilnik	172
150	0.687	0.443	0.657	0.307	0.867	0.318	0.224	0.308	0.664	0.735	0.937	0.184	0.134	121.309	123.991	126.775	135.037	Candelaria	173
4	0.438	0.962	1.000	0.126	0.596	0.343	0.192	0.700	0.530	0.479	0.314	0.184	0.134	4.914	5.518	8.702	15.068	Jacobselv	174
74	0.914	0.898	0.922	0.087	0.914	0.270	0.158	0.709	0.294	0.644	0.429	0.182	0.134	113.693	120.834	127.937	159.299	St. Croix	175
257	0.532	0.705	0.659	0.377	0.413	0.012	0.056	0.624	0.112	0.340	0.415	0.180	0.132	56.967	58.024	58.052	71.070	Serrano	176
204	0.453	0.187	0.229	0.853	0.373	0.352	0.853	0.255	0.368	0.314	0.965	0.179	0.132	73.013	71.153	85.044	83.953	Lotagipi Swamp	177
149	0.564	0.403	0.557	0.550	0.662	0.269	0.458	0.463	0.535	0.693	0.920	0.178	0.131	119.900	120.154	122.313	137.571	Hondo	178
22	0.794	0.439	0.687	0.352	0.615	0.433	0.264	0.597	0.503	0.475	0.436	0.178	0.131	33.026	41.514	47.018	88.089	Narva	179
181	0.190	0.614	0.605	0.494	0.217	0.382	0.355	0.549	0.107	0.963	0.762	0.177	0.131	90.168	94.537	95.792	111.275	Sixaola	180
72	0.527	0.739	0.809	0.211	0.420	0.715	0.499	0.357	0.728	0.650	0.507	0.176	0.130	54.230	56.041	65.278	92.729	Soca	181
264	0.579	0.418	0.654	0.466	0.627	0.000	0.029	0.666	0.575	0.340	0.384	0.176	0.130	62.659	65.489	65.602	75.172	Azopardo	182
260	0.512	0.801	0.661	0.341	0.262	0.090	0.065	0.580	0.022	0.304	0.446	0.173	0.129	30.289	31.493	37.774	46.175	San Martin	183
91	0.346	0.346	0.559	0.690	0.207	0.708	0.518	0.468	0.380	0.650	0.523	0.169	0.127	115.140	122.155	125.039	178.758	Drin	184
67	0.875	0.912	0.914	0.095	0.877	0.327	0.158	0.751	0.385	0.631	0.394	0.167	0.126	82.972	89.305	102.765	171.721	St. John	185
50	0.744	0.838	0.853	0.100	0.800	0.815	0.240	0.195	0.456	0.464	0.516	0.167	0.126	47.848	53.256	56.612	94.477	Elbe	186
27	0.228	0.639	0.679	0.323	0.385	0.478	0.311	0.527	0.465	0.480	0.443	0.164	0.124	20.937	27.914	26.188	79.302	Gauja	187
98	0.712	0.433	0.667	0.552	0.490	0.371	0.318	0.208	0.432	0.434	0.570	0.162	0.123	176.007	172.150	185.032	187.255	Rezovo	188
174	0.363	0.221	0.216	0.810	0.467	0.546	0.643	0.284	0.473	0.573	0.955	0.157	0.121	141.517	144.171	165.421	164.383	Komoe	189
147	0.300	0.186	0.339	0.920	0.363	0.822	0.550	0.130	0.111	0.673	0.867	0.157	0.121	120.411	115.799	118.560	131.013	Artibonite	190
177	0.243	0.170	0.141	0.905	0.454	0.561	0.740	0.460	0.541	0.774	0.948	0.156	0.120	137.920	139.242	152.438	154.596	Great Scarcies	191
219	0.126	0.328	0.640	0.346	0.147	0.166	0.185	0.805	0.496	0.807	0.896	0.155	0.120	173.706	167.770	173.505	187.674	Komo	192
88	0.346	0.414	0.610	0.567	0.210	0.685	0.417	0.333	0.327	0.490	0.556	0.152	0.119	153.223	162.162	162.986	217.157	Vardar	193
16	0.442	0.962	0.998	0.126	0.594	0.424	0.190	0.699	0.411	0.593	0.337	0.151	0.118	16.796	18.906	26.135	40.091	Glomma	194
28	0.262	0.643	0.684	0.350	0.414	0.547	0.319	0.477	0.356	0.492	0.465	0.149	0.117	26.519	29.577	29.132	67.981	Venta	195
25	0.800	0.928	0.905	0.104	0.826	0.000	0.157	0.804	0.380	0.837	0.311	0.149	0.117	18.827	18.859	19.131	31.272	Whiting	196
157	0.339	0.309	0.425	0.612	0.474	0.782	0.578	0.341	0.523	0.652	0.828	0.147	0.116	107.254	108.978	114.931	131.645	Motagua	197
199	0.373	0.419	0.263	0.876	0.307	0.681	0.581	0.344	0.541	0.663	0.950	0.147	0.116	138.030	137.874	160.241	160.190	Tano	198
45	0.760	0.902	0.878	0.000	0.883	0.619	0.159	0.181	0.739	0.540	0.499	0.146	0.115	20.030	17.133	10.005	16.141	Castletown	199
18	0.760	0.933	0.902	0.107	0.807	0.003	0.157	0.790	0.579	0.648	0.212	0.143	0.114	31.921	31.729	30.558	37.024	Alsek	200
85	0.676	0.771	0.840	0.262	0.727	0.503	0.192	0.166	0.426	0.673	0.604	0.143	0.114	80.148	79.947	88.327	144.245	Bidasoa	201
39	0.677	0.905	0.902	0.080	0.724	0.592	0.284	0.349	0.594	0.628	0.495	0.140	0.113	13.628	14.706	17.044	24.157	Foyle	202
168	0.234	0.424	0.439	0.670	0.179	0.694	0.432	0.549	0.205	0.778	0.926	0.139	0.112	171.857	171.636	179.760	192.135	San Juan	203
209	0.541	0.576	0.634	0.532	0.090	0.094	0.338	0.463	0.053	0.928	0.858	0.136	0.111	155.412	158.018	179.530	211.183	Bangau	204
21	0.707	0.938	0.899	0.110	0.787	0.000	0.157	0.804	0.371	0.669	0.278	0.135	0.110	23.876	21.825	20.978	42.618	Taku	205
187	0.237	0.168	0.126	0.929	0.411	0.679	0.724	0.486	0.487	0.814	0.910	0.134	0.110	206.005	203.225	219.225	215.247	Moa	206
32	0.243	0.634	0.679	0.333	0.397	0.438	0.314	0.479	0.406	0.490	0.476	0.124	0.105	23.132	26.922	34.758	72.400	Bartuva	207
52	0.758	0.904	0.893	0.046	0.847	0.903	0.231	0.256	0.506	0.565	0.518	0.124	0.105	65.772	68.285	74.209	108.764	Rhine	208
193	0.538	0.237	0.563	0.517	0.226	0.080	0.204	0.836	0.465	0.810	0.922	0.123	0.105	210.320	210.304	230.299	245.266	Amakuru	209
3	0.451	0.978	0.969	0.163	0.630	0.056	0.170	0.658	0.279	0.410	0.241	0.123	0.104	6.523	4.455	2.202	2.831	Tana	210
65	0.745	0.833	0.871	0.150	0.942	0.735	0.199	0.328	0.556	0.586	0.522	0.121	0.103	73.025	77.041	87.550	126.725	Rhone	211
26	0.232	0.629	0.676	0.325	0.389	0.358	0.312	0.529	0.432	0.470	0.449	0.121	0.103	36.497	42.554	48.109	106.947	Salaca	212
23	0.699	0.939	0.899	0.110	0.785	0.003	0.157	0.803	0.262	0.679	0.264	0.120	0.103	39.127	37.569	35.044	54.081	Stikine	213
176	0.233	0.182	0.125	0.960	0.350	0.590	0.727	0.481	0.510	0.770	0.933	0.117	0.102	195.890	194.008	214.415	211.983	Little Scarcies	214
87	0.655	0.777	0.829	0.256	0.736	0.601	0.249	0.215	0.408	0.466	0.585	0.113	0.099	155.668	158.431	161.268	185.709	Douro	215
29	0.719	0.363	0.663	0.454	0.558	0.500	0.269	0.536	0.495	0.479	0.443	0.113	0.099	73.402	86.984	85.046	140.055	Daugava	216
60	0.754	0.821	0.865	0.158	0.951	0.866	0.196	0.333	0.316	0.490	0.567	0.112	0.099	107.133	106.555	113.580	167.160	Seine	217
96	0.657	0.776	0.830	0.256	0.735	0.601	0.244	0.282	0.569	0.661	0.570	0.111	0.099	111.822	115.256	136.297	229.442	Lima	218
75	0.754	0.821	0.865	0.158	0.951	0.639	0.196	0.331	0.484	0.509	0.594	0.111	0.099	141.552	148.393	151.884	178.245	Garonne	219
186	0.213	0.248	0.129	0.890	0.305	0.691	0.725	0.317	0.402	0.633	0.946	0.106	0.096	212.271	221.510	247.575	251.098	Mono	220
78	0.754	0.821	0.865	0.158	0.951	0.349	0.196	0.116	0.456	0.610	0.411	0.104	0.095	8.357	15.140	18.320	97.793	Roya	221
100	0.701	0.425	0.656	0.565	0.471	0.539	0.334	0.241	0.524	0.646	0.437	0.102	0.094	161.495	173.102	193.599	264.468	Coruh	222
34	0.468	0.444	0.668	0.482	0.476	0.617	0.295	0.405	0.437	0.458	0.469	0.102	0.094	90.623	95.664	99.557	153.148	Neman	223
200	0.185	0.510	0.633	0.391	0.217	0.097	0.371	0.729	0.085	1.000	0.883	0.100	0.093	231.996	231.870	230.575	289.672	Jurado	224
83	0.673	0.772	0.838	0.261	0.729	0.602	0.201	0.175	0.519	0.650	0.568	0.100	0.093	108.232	108.621	117.314	186.477	Minho	225
9	0.540	0.989	0.884	0.168	0.558	0.141	0.108	0.535	0.423	0.455	0.263	0.097	0.092	17.455	13.514	10.072	23.592	Torne	226
103	0.408	0.437	0.643	0.705	0.442	0.605	0.489	0.412	0.320	0.688	0.584	0.097	0.091	208.022	216.359	230.081	254.334	Vjose	227
17	0.549	0.973	0.913	0.127	0.474	0.493	0.117	0.568	0.358	0.519	0.412	0.096	0.091	44.189	53.186	65.902	110.718	Gota alv	228
160	0.253	0.329	0.327	0.701	0.185	0.542	0.486	0.524	0.592	0.749	0.866	0.093	0.090	193.171	193.212	195.294	205.530	Coco	229
5	0.466	1.000	0.917	0.207	0.671	0.045	0.140	0.586	0.314	0.444	0.271	0.093	0.090	−2.885	−3.258	−3.179	0.967	Naatamo	230
6	0.600	0.943	0.880	0.240	0.687	0.081	0.138	0.571	0.316	0.428	0.271	0.091	0.088	13.682	13.299	10.993	23.334	Paatsjoki	231
180	0.365	0.171	0.225	0.780	0.487	0.642	0.606	0.323	0.618	0.662	0.917	0.091	0.088	232.020	231.195	268.440	268.096	Sassandra	232
190	0.224	0.123	0.070	0.917	0.401	0.582	0.664	0.519	0.507	0.786	0.898	0.084	0.085	310.934	308.310	311.350	299.824	Saint Paul	233
226	0.441	0.328	0.154	0.860	0.474	0.507	0.875	0.321	1.000	0.494	0.884	0.082	0.084	192.595	190.027	207.611	215.557	Umba	234
198	0.234	0.113	0.017	0.970	0.183	0.534	0.593	0.538	0.698	0.775	0.918	0.081	0.084	324.308	316.243	308.188	327.404	Cestos	235
11	0.565	0.974	0.870	0.242	0.694	0.134	0.128	0.549	0.438	0.443	0.295	0.080	0.083	15.783	15.669	15.313	51.772	Kemijoki	236
208	0.626	0.473	0.577	0.579	0.118	0.256	0.431	0.441	0.182	0.919	0.896	0.077	0.082	271.152	272.996	278.096	325.800	Sembakung	237
191	0.202	0.111	0.000	0.968	0.236	0.524	0.617	0.560	0.469	0.841	0.902	0.074	0.080	317.685	315.511	317.048	313.667	Lofa	238
37	0.760	0.902	0.878	0.000	0.883	0.786	0.159	0.181	0.653	0.568	0.513	0.074	0.080	−13.186	−11.370	−13.433	2.049	Bann	239
14	0.567	0.973	0.870	0.242	0.694	0.245	0.128	0.546	0.570	0.445	0.344	0.069	0.078	21.673	28.563	33.546	144.646	Oulujoki	240
202	0.373	0.339	0.253	0.848	0.374	0.661	0.582	0.325	0.531	0.661	0.950	0.066	0.076	229.932	228.168	260.177	259.918	Bia	241
197	0.206	0.113	0.016	0.959	0.279	0.600	0.626	0.559	0.569	0.764	0.911	0.066	0.076	355.824	352.873	341.956	351.122	Saint John	242
38	0.665	0.968	0.888	0.105	0.633	0.747	0.134	0.219	0.448	0.531	0.511	0.058	0.072	37.020	38.330	54.346	113.547	Vida	243
156	0.345	0.308	0.431	0.608	0.490	0.580	0.580	0.327	0.671	0.853	0.910	0.045	0.066	264.224	265.564	278.304	310.664	Sarstun	244
53	0.660	0.844	0.868	0.083	0.804	1.000	0.015	0.199	0.328	0.516	0.554	0.044	0.065	99.300	104.848	123.365	190.222	Scheldt	245
167	0.248	0.332	0.316	0.715	0.161	0.568	0.466	0.554	0.198	0.729	0.916	0.042	0.065	364.270	361.837	339.826	352.057	Negro	246
195	0.201	0.138	0.023	1.000	0.090	0.524	0.637	0.546	0.438	0.864	0.915	0.036	0.062	411.293	404.598	422.166	459.592	Mano	247
47	0.760	0.902	0.878	0.000	0.883	0.742	0.159	0.181	0.706	0.564	0.511	0.025	0.056	−12.873	−10.599	−17.494	−1.670	Flurry	248
207	0.582	0.179	0.202	0.840	0.495	0.265	0.644	0.357	0.515	0.906	0.935	0.020	0.054	412.128	407.433	470.367	462.026	Akpa Yafi	249
54	0.648	0.846	0.868	0.076	0.787	0.824	0.000	0.297	0.286	0.497	0.553	0.001	0.044	60.077	80.453	111.309	260.706	Yser	250
196	0.324	0.146	0.157	0.880	0.390	0.542	0.597	0.473	0.700	0.746	0.911	0.000	0.044	546.906	535.628	538.646	589.893	Cavalla	251

## Annex A Data specification and source

### IRCC and TFDD water events databases

The Transboundary Freshwater Dispute Database (TFDD) International Water Event Database (IWED) (Wolf et al., 2003; Yoffe et al., 2003, 2004; De Stefano et al., 2010b) provides information about international water basin interactions between 1948 and 2008; the International River Cooperation and Conflict database (IRCC), reports water related issues between 1997 and 2007 (Kalbhenn and Bernauer, 2012). Both databases are set up in the form of water related events at dyad-basin level. Each national territorial unit in a specific river basin is defined as a basin-country unit (BCU), each of the possible pairs of BCU’s in the same basin are classified as a dyad. Water related events (or interactions) are classified in the basis of a scale assigning a score representing the intensity of the issue, and its nature (conflict/cooperation): +6 most cooperative, -6 most confrontational in the IRCC case (Kalbhenn and Bernauer, 2012); -7/+7 in the TFDD case (Yoffe et al., 2004; Yoffe and Larson, 2002). The interactions related to the same water issue involving two or more BCU’s are clustered in a specific “case” (multiple events could be attributed to each water case), representing the hydro-political issue determining the interactions between the countries sharing a watershed. Although the temporal coverage (11 years) is limited, the IRCC database was preferred in this analysis for the higher number of non-neutral interactions reported.

Due to the nature of the algorithms used for the creation of the database, mining water coded events from international news datasets, the event data are characterized by an uneven geographical distribution of the observations. About 4800 of the 5881 observed events refer to the most represented international river basins, namely Danube, Nile, Zambezi, Mekong, Euphrates/Tigris, Ganges/Brahmaputra, Aral Sea, Elbe/Labe (Kalbhenn and Bernauer, 2012). In total, the IRCC dataset counts 15965 entries (5881 events and 10084 combinations of dyad countries-basin-year with no events), it presents data about 262 transboundary basins, 760 dyads countries, and 1279 combinations basin/dyads (respectively 261, 725, and 1249 for the TFDD dataset) (Kalbhenn and Bernauer, 2012). Due to data limitation, 11 dyads and 2 minor basins (total of 43 observations, 21 of which non-zero)^11^ were excluded.

### Alternative water events databases

Alternative event datasets are currently available, namely: the Water and Conflict Chronology (Gleick, 1998; Gleick et al., 1994)^12^, a collection of historical conflicts where water was the object of the dispute, a side political goal, a military tool, or object of terroristic attacks; the Water-Related Intrastate Conflict and Cooperation (WARICC) (Bernauer et al., 2012a), providing information about water related events in 35 countries in the Mediterranean, Middle-East, and Sahel areas; and the Issue Correlate of War – River Claims Data Set (ICOW-River), collecting river management issues data about 82 dyads in 36 rivers mainly in the Western Hemisphere and Middle East (Hensel et al., 2008, 2006). The mentioned alternative databases were not taken into consideration in this analysis due to respectively the bias potentially rising from the un-homogeneous data collection methodology (in the case of Water and Conflict Chronology), and the limited geographical coverage (in the cases of WARICC and ICOW-River). Moreover the WARICC dataset is a collection of domestic water tension/cooperation events, which only in part overlaps the domain of the transboundary interactions object of the analysis hereby presented. However, in this study, the WARICC dataset was still considered as a source of information about the spatial distribution of water management critical hotspots within the selected basins.

### Standardized precipitation Index

The index is a measure expressed in standard deviation units of the variation of the precipitation of a specific number of months respect to the long run average (WMO, 2012). The number of months based on which the SPI could be calculated usually varies between 3 and 48 months. Shorter time scales SPI is considered a good indicator of variations of soil moisture, while on longer scales (up to 24 months), it could be associated with groundwater or reservoir levels variation (WMO, 2012). That for, a *shorter* SPI (3 months) is often utilized to detect meteorological droughts; a *medium* SPI (6 months) is usually associated with agricultural droughts; a *longer* SPI (12–24 months) is associated with hydrological droughts. SPI was calculated using the R package SPEI (Beguería and Vicente-Serrano, 2014).

### Climate modeling outputs - projections used in the study

Climate projections data used in this study belong to the NASA Earth Exchange Global Daily Downscaled Projections (NASA NEX-GDDP) dataset downscaled (0.25 degrees) and bias corrected using the Bias-Correction Spatial Disaggregation (BCSD) methodology described in Thrasher et al. (2012). NEX-GDDP includes all the 21 GCM’s built in support of the Fifth Assessment Report of the Intergovernmental Panel on Climate Change (IPCC AR5). Due to computational constraints, we selected 5 out of the 21 climate models, chosen in base of the structural differences among them, as described in Knutti et al. (2013). In particular, in this study the following GCM’s were chosen: the National Center for Atmospheric Research (NCAR) Community Climate System Model (CCSM) version 4.0 (NCAR CESM, 2011); the National Oceanic and Atmospheric Administration (NOAA) Geophysical Fluid Dynamics Laboratory (GFDL) Coupled Model (CM) version 3.0 (Donner et al., 2011); the Institute for Numerical Mathematics (INM) Climate Model (CM) version 4.0 (Volodin et al., 2013, 2010); the Institut Pierre-Simon Laplace (IPSL) Climate Model (CM) version 5.0 A – Medium Resolution – (Dufresne et al., 2013); and the Model for Interdisciplinary Research on Climate (MIROC) version 5.0 (Watanabe et al., 2010).

## Annex B Random Forest regression algorithm

RF is a Classification and Regression Tree (CART) based tool that involves an ensemble of regression trees (Breiman, 2001). The estimated trees, each of them being a regression model fit using a subset of the input data and a portion of the independent variables, are then averaged in order to reduce the bias typical of the bagging (bootstrap aggregating) techniques (Breiman, 1994). Each of the tree is a noisy but virtually unbiased regression model: the aggregation procedure allows to reduce the variance (Hastie et al., 2009).

The RF regression algorithm procedure could be summarized in few steps:

A A random subset of the training data is drawn (each tree is trained using about 63%, ∼2/3, of the initial observations);
B For each of the bootstrapped subsets, a tree is grown by recursively repeating three actions: select a random subset of the independent variables (*m<p,* being *p* the total number of the independent variables); calculate the best variable/split among the *m* variables; generate two sub-nodes. This iteration is repeated until the minimum number of observation per node is reached. Each tree is tested against the remaining observations (about 37%) and the Out-of-the-Bag error (OOB – the mean prediction error on each training subset) is calculated (specifically, in the case of RF regression, the Mean Squared Error – MSE (Eq. 1))
C Aggregate the generated trees in an ensemble and calculation of the overall MSE (Hastie et al., 2009).

The model is tuned through the calibration of three parameters: number of trees (*ntree*); the number of variables per node (*mtry*); and the minimum number of observations for the final node, often called leaf (*nodesize*) (Li et al., 2016; Malekipirbazari and Aksakalli, 2015). RF error is determined by mainly two factors: correlation between the trees; and strength of the individual tree. The *m* number of randomly selected independent variables, by default chosen as a third of the whole number of regressors (*p*), is crucial for this. In fact, a larger *m* (*mtry*) value would increase the correlation between the trees, while increasing the trees strength, and vice-versa (Breiman, 2001). Increasing the number of trees (*ntrees*) would stabilize the model and reduce the overall error, until reaching a point in which the correlation between the trees would start to rise, consequently decreasing the overall model performance. Given its characteristics, RF is almost insensitive to tuning in the size of the final node (*nodesize*), and consequently to the length of the tree (Segal, 2004). RF is particularly effective in capturing complex non-linear relations; the model can handle a certain degree of multicollinearity between the dependent variables; it is almost completely immune from overfitting; it is insensitive to outliers, and does not require data pre-processing (Hastie et al., 2009). Moreover, RF is not sensitive to monotonic transformations of the independent variables; at the same there is no need to perform a feature selection: RF automatically ignores the variables that do not ensure a good split. This model was successfully applied in many fields of study in which the traditional statistical analysis is afflicted by the problem of multicollinearity and the independent variables are characterized by high covariance, as for instance: genomics (Chen and Ishwaran, 2012), remote sensing (Jing et al., 2016; Rasquinha and Sankaran, 2016; Vogels et al., 2017), public health (Loidl et al., 2016), hydrology (Li et al., 2016; Mohr et al., 2017; Núñez et al., 2016), agriculture (Jeong et al., 2016), and ecological indicators (Pourtaghi et al., 2016). To our best knowledge, the assessment presented in this paper is the first application of a RF approach to a dyadic dataset in the context of international water interactions.

Since it is performed internally while estimating the OOB error, RF does not need cross-validation. However, as in previous studies (Jeong et al., 2016; Li et al., 2016; Malekipirbazari and Aksakalli, 2015), we performed a 10 fold cross validation to test the performance of the RF model in comparison with alternative algorithms, namely: Generalized Linear Regression (GLM), Boosted Decision Trees (BDT), and Support Vector Machine (SVM). The model performance was compared calculating the coefficient of determination *R^2^* (Eq. 2) and the Root Mean Squared Error *RMSE* (Eq. 3).

(1) $$MSE=\;\sum_{i=1}^{n}{\left(y_{i}-\;{\hat{y}}_{i}\right)}^{2}\;/\;n$$

(2) $$R^{2}=1-\;\frac{\sum_{i=1}^{n}{\left(y_{i}-\;{\hat{y}}_{i}\right)}^{2}\;}{\sum_{i=1}^{n}{\left(y_{i}-\;{\bar{y}}_{i}\right)}^{2}\;}$$

(3) $$RMSE=\;\sqrt{\sum_{i=1}^{n}{\left(y_{i}-\;{\hat{y}}_{i}\right)}^{2}\;/\;n}$$

Being $y_{i}$ the observed values, ${\hat{y}}_{i}$ the modeled, and ${\bar{y}}_{i}$ the observation mean.

The final calibrated RF model was trained using the entire set of observations (*N*=11,801). Model performance was estimated calculating MSE (Eq. 1) and pseudo R^2^ (Eq. 4), a measure of the variation explained by the model (Kvålseth, 1985; Seber and Lee, 2003).

(4) $$pseudo\;R^{2}=1-\;\frac{MSE}{Var(y)}$$

Where MSE represents the Mean Standard Error (Eq. 1), and Var(y) represents the variance of the observed values.

An additional important feature of RF is the possibility to quantify the relative importance of each of the explanatory variables by estimating the MSE variations when a specific independent variable is permuted (Breiman, 2001; Hastie et al., 2009). Given the random nature of the model, variable relative importance is rather volatile. Although, more stable values could be achieved if the number of trees is sufficiently high, variable importance is likely to vary within a certain range especially in case of correlated variables (Altmann et al., 2010). That for, variable ranking could virtually experience permutations every time a RF model is performed (Hastie et al., 2009; Strobl et al., 2007). In order to avoid this problem, our final tuned model was run 100 times recursively and the variable relative importance estimates were presented in a boxplot form (Fig. A3). Variable interactions and final results of the model were presented using 2-dimensional and 3-dimensional partial dependency plots (Hastie et al., 2009; Welling et al., 2016). The analytical experiment was performed using the statistical software *R* in combination with the packages: *randomForest* (Liaw and Wiener, 2002), *forestfloor* (Welling et al., 2016), *caret* (Kuhn, 2008), and *varSelRF* (Diaz-Uriarte, 2014).

In order to validate our choice in terms of methodological approach, we tested the RF model performance in comparison with alternative statistical approaches, one linear model and two other algorithms derived by machine learning. In the 10-fold cross validation, the RF model outperformed the alternative methods by minimizing the error (mean RMSE = 0.218) and maximizing the coefficient of determination (mean R^2^=0.679). The Generalized Linear Model (GLM) was the least performant, followed by the Boosted Decision Trees (BDT), and Support Vector Machine (SVM) (Fig. A1). In order to ensure comparability, all the statistical approaches were tuned with a multiple-steps procedure and the best performant parameters were selected for the final assessment.

RF parameter calibration was performed running the model recursively minimizing the Mean Squared Error (MSE) and maximizing the variation explained (pseudo R^2^). Increasing the number of trees rapidly stabilized (*ntree*∼150) the MSE below 0.05: the performance marginal gain for values above this *ntree* was found almost completely negligible. However, the final setting of this parameter was set to 500 to give more robustness to the model. The number of variables randomly selected per split (*mtry*), by default set at 6 (∼*p/3*), was found to be optimal at a value of 8. While the minimum number of observations for the final node (*nodesize*) value that minimized the error was found to be the default one (2 observations) (Fig. A2). This confirms the theoretical literature indicating that the selected model handles comfortably fully grown trees without overfitting.

## Annex C Comparison with the spatial distribution of the water interaction recorded in the WARICC database

Our analysis highlights the areas of the transboundary basins where water interactions are more likely to rise. As an empirical validation of our results, we compared the spatial distribution of our index with geospatial data about water interactions. The only dataset of historical water related events available at fine (sub-dyadic) resolution is the Water-Related Intrastate Conflict and Cooperation (WARICC) dataset (Bernauer et al., 2012a), classifying water events recorded in 35 countries in the Mediterranean, the Middle East, and the Sahel in the period 1997–2009. A comparison between the spatial distribution of the water events classified in the WARICC dataset and the here presented likelihood of hydro-political interactions is presented in Fig. A6. It should be noted that our index was structured studying the transboundary water interactions, while the WARICC database is a repository of domestic conflict and cooperation events over water. The spatial distribution of the events corresponds to the highest values of our index for the majority of the areas under consideration, especially within the boundaries of the main transboundary river basins. The correspondence of the presented index and the events outside the boundaries of the main river basins, especially in the Adriatic portion of the Balkans, is less evident. However, there is the possibility that the high concentration of the WARICC reported events in the Balkan area could be linked with the civil conflicts that followed the collapse of the Socialist Federal Republic of Yugoslavia (1992, with the civil war spanning a decade between 1991 and 2001). Similar bias could affect the WARICC event data at the border between Eritrea and Ethiopia involved in a territorial dispute between 1998 and 2000.

## Footnotes

1 An overview about this topic is provided, among others, by the Correlates of War Project (http://www.correlatesofwar.org/)

2 http://www.sdg6monitoring.org/indicators/target-65/indicators652/

3 www.transboundarywaters.orst.edu; http://gis.nacse.org/tfdd/index.php

4 http://cow.dss.ucdavis.edu/data-sets/national-material-capabilities/national-material-capabilities-v4-0

5 www.transboundarywaters.orst.edu

6 *TOT_Precip*, *MIN_Precip*, *SPI_12*, *AVG_Temp*, *TempMAX*, *TempMin*, *Temp_delta*, *Temp_seasonal_var* in Table A1.

7 *Pop_density* in Table A1.

8 *Falkenmark_upst* in Table A1.

9 *Rural_pop, GDP, Agriculture, Governance_ind, cinc_mean, IFTD_treaties* in Table A1.

10 With a maximum of 166 interactions between Hungary and Romania in the Danube river basin in the year 2000.
